# Adolescent Deviance and Cyber-Deviance. A Systematic Literature Review

**DOI:** 10.3389/fpsyg.2021.748006

**Published:** 2021-10-12

**Authors:** Smaranda Cioban, Adela Răzvana Lazăr, Claudia Bacter, Adrian Hatos

**Affiliations:** ^1^Faculty of Social Humanistic Studies, Doctoral School of Sociology, University of Oradea, Oradea, Romania; ^2^Faculty of Social Humanistic Studies, Psychology Department, University of Oradea, Oradea, Romania

**Keywords:** deviance, cyber-deviance, adolescents, predictors of deviance, systematic (literature) review, KH coder

## Abstract

Deviance is a complex phenomenon that influences aspects both at the macro and micro levels, extensively studied by social scientists The main objective of this article was to conduct a systematic literature review for clustering the topics on adolescent deviance and online deviance. Grounded in Pickering's and Byrne's guidelines and PRISMA protocol, we identified the most recurrent themes, theories and predictors in the 61 most-cited articles related to the concept of deviance from the database of Web of Science, as well as in 488 abstracts of representative papers. The results emphasized four main clusters of topics, namely, predictors of deviance, online deviance, socio-constructivist theories, and research based theories of deviant behavior. The findings highlighted that researchers frequently use strain theory, social learning, self-control, and social control theories in their studies. Our systematic literature review revealed also the most encountered predictors of deviance, which we have classified into five main categories: family patterns, socio-demographic aspects, socialization, victimization, and school and individual factors. For online deviance, family patterns, socio-demographic aspects, victimization, school and individual factors, and Internet and computer use have been determined to be the main groups of predictors. The present systematic literature review makes an important contribution to the understanding of deviance by presenting an overview of the phenomenon.

## Introduction

From a multidisciplinary view, deviance is a topic of interest for social scientists as it concerns the violation of approved rules and established norms. As such, there is an abundance of published studies on this research topic, which, however, makes the process of understanding the phenomenon quite challenging.

Consistent with the age-crime curve approach (Moffitt, 1993b; Shulman et al., 2013; Farrington, 2017) which sustains that teenagers engage into deviant behaviors more often than other categories, adolescence is worth considering in studying deviance. In addition, with the spread of Internet technology, a new type of deviant behavior emerged, which is known as cyber-deviance, particularly prevalent in adolescents' lives. As such, teenagers' online deviant behavior has become a matter of grave concern for parents, educators, and researchers.

### The Concept of Deviance

The phenomenon of deviance occupies a central position in social science topics. It may be linked to morality, social order, and social inequality. Its genesis has been traced to the 1940s and is credited to Merton (1938) and Sellin (1938), scholars belonging to the Chicago School of Sociology. They defined deviance as a topic of analysis of socio-criminogenesis, a field that encompasses research in criminology, psychiatry, psychology, and sociology.

The wide range of fields interested in defining and studying deviance shows the level of complexity of this phenomenon. As Parsons (1951) emphasizes, deviance affects both the individual and the macrosystem, characterized by deviation from the moral and established rules that form the mainstream culture. According to the author, on an individual level, deviance represents the motivated tendency of an actor to behave in contradiction of one or more institutionalized social rules (Parsons, 1951). The behavior at the individual level has consequences at the social level. From a macro perspective, deviance is the opposite of social control. Hence, it represents the tendency to disbalance the social system, while social control mechanisms act to reestablish social equilibrium.

Social disapproval is an illustrative example of a social control mechanism. Therefore, deviant acts are not just rule-breaking, they are also behaviors that are against the societal or communal norms, which may trigger social disapproval (Clinard and Meier, 2015; Goode, 2016). Social disapproval is expressed by a lack of acceptance, isolation in a community, and social sanctions such as shame and guilt, which end up being accepted by community members as well as the individual as legitimate (Elias, 1978).

The mechanism of internalizing shame and guilt as a disciplinary practice is also a focus of Foucault's (1971) perspective that portrays deviance as normal and regulatory in society. With regards to presenting institutionalization and confinement as instruments to impose the rules of the dominant groups, Foucault (1971, 2007) explains how the external discipline is transferred to a discipline that is imposed from within. An internalized, self-disciplinary power is under the threat of being exposed. From this perspective, deviants seem to be the marginalized ones, the ones to lose their power to hold onto their own values and are forced to accept societal norms through isolation, surveillance, and discipline. The individuals committing rule-breaking, deviants, may be disciplined harshly and, therefore, eventually become a non-deviant (Foucault, 1971, 2007; Turkel, 1990). Complementary to Foucault's view, Durkheim (1938) characterizes deviance as being inevitable, present in any society, and necessary for its functioning.

### Theories of Deviance

In the quest to understand the phenomenon of deviance, the question of whether deviant acts and, therefore, deviant individuals are born or is society the one that labels them as such arises. This is a central topic in the study of deviance, with essentialist and positivist approaches arguing that deviant individuals are born with specific traits that influence their behavior, whilst constructivist scholars contend that society marks individuals as deviant (Thio et al., 2013).

To expand on their idea of the presence of deviant traits in individuals, positivists attempted to identify specific characteristics of this phenomenon, which resulted in the formulation of many theories. The most common *positivist theories* include social learning theories (Bandura, 1978; Akers and Lee, 1999; Akers, 2017), strain theory (Cohen, 1955; Cloward, 1959), anomie theory (Merton, 1938), self-control theory (Akers, 1991; Hirschi and Gottfredson, 2000), deterrence theory (Gibbs and Erickson, 1975; Warr and Stafford, 1991), differential association theory (Sutherland et al., 1992). The inclination of an individual toward engaging in deviant acts is what distinguishes a deviant from a non-deviant. This framework traces back from the sociobiological, psychological, and criminological research of Italian naturalists who attempted to identify biological features or psychological traits unique to deviants (Lombroso-Ferrero, 1911). The biological orientation has now promoted more sophisticated approaches, assuming the presence of specific genes or genomic segments as proof of inclination toward addictive and risk-taking behaviors (Shostak et al., 2009; Linnér et al., 2019; Mills and Tropf, 2020). These studies belong to sociogenomics, a discipline that connects genetics and sociology (Udry, 1995; Duster, 2006a,b; Mills and Tropf, 2020).

While positivist theorists claim that an act is seen as deviant because it breaks the norms of a particular society, constructivists notice that some acts are perceived as deviant only in a particular context but are not universally categorized as deviant and distinguished between deviance and crime. The most relevant *constructionist theorists* include labeling theories (Erikson, 1962; Lemert, 1967; Ben-Yehuda, 1990; Becker, 1995, 2008), symbolic interactionism (Clinard and Meier, 2015), phenomenological theories (Matza and Blomberg, 2017), and social conflict theories (Foucault, 1971; Jensen et al., 1978; Mills, 1981; Hagan et al., 1985; Katz, 1988; Henry and Milovanovic, 1996; Milovanovic, 1996; Hagan and McCarthy, 1998). Constructivists argue that it is not the act that is deviant but society's act of labeling it as such that makes it deviant. In this sense, one has to acknowledge the role of cultural differences in labeling an act as deviant (Goffman, 1978; Clinard and Meier, 2015), although criminal acts are universally defined as deviant. Deviance is relative as it depends on the context in which it is judged and on how society labels a particular act or individual. Moreover, deviance is the result of a subjective experience, in that each person provides a certain meaning to the acts they are involved in. At the same time, deviance is voluntary, being regarded as an expression or choice of a person (Erikson, 1962; Ben-Yehuda, 1990; Becker, 2008).

In an *integrative approach*, Thio et al. (1978) argue that the two above-mentioned frameworks are complementary. Therefore, the authors distinguish between higher consensus deviance and lower consensus deviance. Higher consensus deviance includes acts that are generally perceived as deviant and cause major losses, while lower consensus deviance relates to acts that are seen as deviant by fewer persons because those acts cause minor losses (Thio et al., 2013).

The classification of deviant theories is related to many research areas. The present study focuses on the psychological and sociological aspects as they relate to deviance in teenagers. Specifically, *psychologists* analyze deviance from the point of individual characteristics. The main cause for deviant behavior is found strictly at the individual level and, in our case, at the adolescent stage. The most important psychological theories refer to psychoanalytic theories, such as, Freud's (2012), the cognitive development theory of Kohlberg and Hersh (1977), and the learning theory (Bandura and McClelland, 1977). Freud believes that all children are born with tendencies toward deviant behaviors, but appropriate socialization experiences can help them not become deviant. On the other hand, Kohlberg and Hersh (1977) claimed that there exist stages of moral development, with deviant adolescents being those who have failed to pass through the pre-conventional and conventional stages of moral development. According to Bandura, deviant behavior is learned by observation, imitation, and shaped by rewards and punishments (Bandura, 1971, 1978; Bandura and McClelland, 1977).

On the other hand, *sociologists* study the emergence of deviance and its impact on societies, communities, or groups and develop theories that view the phenomenon on a wide scale (Bandura, 1971, 1978; Bandura and McClelland, 1977). Hirschi's theories of social control (Hirschi, 1969, 2017; Pratt and Cullen, 2000), the theory of differential association (Sutherland et al., 1992; Ogien, 2002), and Glaser's theory of differential identifications (Erikson, 1962; Glaser, 1971; Rowitz, 1981) present deviance as a result of group socialization. Considering the ideas of these orientations, deviance in teenagers is associated with family factors, peer group pressure, and school climate. Another perspective links deviant acts to status frustration. Thus, Cohen's subcultural theory contends that teenagers' deviant behavior is a tactic used by those with low social status and unsatisfactory social conditions to attain a higher social status in a short time (Cohen, 1955, 2002; Barmaki, 2016). In addition, structural theorists view socioeconomic status as a predictor of deviant behavior, frequently encountered in middle-class teenagers (Hagan et al., 1985; Hagan, 1990; Hagan and McCarthy, 1998; Hagan and Foster, 2001). Therefore, deviance is a result of the antinomy between means and social aspirations, which is also a topic discussed by the anomie theory, with anomie being defined as the state of society characterized by a lack of norms and social values (Merton, 1938). Socio-constructivist theories that present deviance as a result of social judgment and labeling also have sociological orientations (Becker, 1995, 2008; Yoder, 2011).

### Types of Deviance

Along with formulating the theories to explain the general tendency of deviance, scholars have also distinguished specific types of inappropriate behavior. Deviance behavior ranges from serious offenses, classified as delinquent acts (such as property crime, violent crime, delinquency, drug and substance-related crime, white-collar crime, etc.) to minor antisocial acts that are not sanctioned by the penal system (Gorman-Smith et al., 1998).

With regards to adolescents' engagement in deviant behaviors, scholars focused on substance and alcohol use (Durkin et al., 2005; Maimon and Browning, 2012b), marijuana use (Winfree and Griffiths, 1983; Akers and Cochran, 1985; McBroom, 1994; Ennett et al., 1997), school misconduct (Musgrave, 1980; Lepoutre, 2007; Peguero, 2011)), self-injury (Adler and Adler, 2007; Brossard, 2014; Taylor and Ibanez, 2015; Long, 2017), self-harming behaviors such as eating disorders (Sischo et al., 2006), bullying (Juvonen and Graham, 2014; Tippett and Wolke, 2014).

A particular category of deviance, increasingly common nowadays, is *cyber-deviance*, which refers to the harmful activities happening in the digital sphere (Jewkes and Yar, 2013; Graham and Smith, 2019; Yar and Steinmetz, 2019). While the current understanding of deviance is rooted in Durkheim's (2002) definition of crime, the debate on cyber deviance (online deviance) can be traced back to Wall's (2001) conceptualization of cybercrime. According to Wall (2001, p.2), it refers to an “occurrence of a harmful behavior that is somehow related to a computer, which generates a powerful response from the media, policy-makers, politicians, academics and the public.” This definition outlines two main characteristics of cybercrime, namely, the electronic environment and the impact related to the increased concern for cybersecurity. Arguing that Wall's definition of cybercrime is too broad, Yar and Steinmetz (2019) conceptualized cybercrime as a range of illicit activities related to information communication technologies. Therefore, cybercrime refers to online harmful activities sanctioned by formal laws. The other types of online disruptive activities are included under the umbrella term of cyber deviance, which relates to informal violations of laws (Jewkes and Yar, 2013; Graham and Smith, 2019; Yar and Steinmetz, 2019).

Nevertheless, like in the case of crime and deviance, there is no clear demarcation between cybercrime and cyber deviance. This is because norm-breaking behaviors may be included in the formal regulations at any time and certain behaviors that are sanctioned by law may not be regarded as deviant in all contexts. In the study of cyber deviance as occurring in the adolescent stage, scholars mostly focus on digital piracy (Udris, 2016), online harassment and computer hacking (Lee, 2018), cyberbullying (Hinduja and Patchin, 2010; Holt et al., 2012; Lee, 2018), sexting and online sexual exposure (Karaian, 2012; Garcia-Gomez, 2017; Dodaj et al., 2020).

The phenomenon of deviance has an extensive volume of literature that is organized in disparate clusters of studies on various specific types that arouse social concern on the one hand and a preoccupation with in-depth accounts of theoretical approaches on the other. Although extremely rich, the existing literature is narrow in terms of its focus, and there is a need for a more structured presentation of common topics by researchers in the field from a systematic point of view. Most of the reviews that follow a perspective rooted in social sciences (psychology, sociology, criminology, communication) relate to positive deviance (Albanna and Heeks, 2019; Alzunitan et al., 2020), workplace deviance (Götz et al., 2019; Arshad and Malik, 2020), and substance use. Other types of deviance under scrutiny are different types of aggression, antisocial disorders in adolescents, sexual offenses, sexual deviance, social deviance, white-collar crimes, delinquency, and cybercrime. With regards to the reviews conducted on the topic of deviance, the main topics of interest are specific forms of offline deviance, namely, drug use, alcohol abuse, violence, dating violence, sexual aggression, deviant sexual fantasies, illicit sexual behaviors, sexual deviance, ritualistic child abuse, pornography exposure, self-injury, etc. At the same time, researchers focus on different types of online deviance, such as cyberbullying (including adolescent cyberbullying), Internet-based radicalization, online sexual deviance, online negative user behavior, cyber dating abuse, social spamming, problematic social media usage, and problematic use of the Internet.

As far as our knowledge, there is no systematic review that covers the main theories of deviance from a psychological and sociological (including criminological) perspective. Still, comprehensive classifications of these theories are present in textbooks (Thio et al., 2013) and classical studies (Sagarin and Montanino, 1976; Short and Meier, 1981; Sampson and Laub, 1992; Birkbeck and Lafree, 1993; Moffitt, 1993b; Feinberg, 2011). Focused on examining the literature regarding the human reinforcement learning process, the systematic review of Brauer and Tittle (2012) presents the results of a comprehensive analysis of 179 experimental sources and 67 peer-reviewed journal articles. Other theories related to deviance covered in the literature reviews are social learning theory, social control theory, differential association theory, life-course perspectives, social disorganization, strain theory, subcultural theory, social concern theory, routine activities theory or situational approaches, lifestyle exposure theory, arousal theory, criminology's situational approach, rational choice, delinquent problem-solving, deviance regulation theory, interactionist conception, neo-cognitive learning theory, gene-based evolutionary theory, desistance theories, neutralization theory, frustration theory, etc. In the majority of the literature reviews, deviance was not studied as a single phenomenon, but as it related to other factors such as religiosity (Adamczyk et al., 2017), family influence (parental communication (Roisko et al., 2014), parental styles (Ruiz-Hernández et al., 2019), parental control, family processes, family history of substance use), peer-related factors (peer influence (Leung et al., 2014), peer network, peer association, motivations of dissent in social groups), individual factors (animal cruelty (Chan and Wong, 2019a,b; Longobardi and Badenes-Ribera, 2019), victimization and sexual victimization [(McGrath et al., 2011; Dennis et al., 2012; Engström, 2021), child maltreatment (Fitton et al., 2020), non-emotional callousness and impulsivity (Toro et al., 2020), motivational processes (Agnew, 1995)]. Concerning online deviance, most of the systematic reviews refer to a specific type of behavior, which is cyberbullying (Kowalski et al., 2014; Watts et al., 2017; Vale et al., 2018; Rosa et al., 2019; Zych et al., 2019). While reviews on online deviance focus on main theories of deviance, the systematic reviews in the field of online literature focus on specific online behaviors and their correlates, including the predictors of offline deviance mentioned before, Internet access, and computer use. Of particular interest is the systematic review of Estévez et al. (2020), which provides a bridge between online and cyber-deviance by revealing similar patterns in the development of bullying and cyberbullying behavior. In this sense, the authors show that risk factors and protective factors of the two problematic behaviors mostly coincide.

Grounded on the previous analyses conducted in this field, our present systematic literature review inquires the following: *How are the topics of deviance and online deviance covered in the in the field of social sciences?*

In consideration of our question, the goal of this systematic review is to collate and summarize the literature on the field of deviance and online deviance, with a particular focus on teenagers' behaviors, and to achieve this purpose, the article proposes specific *objectives* in order to go beyond a panoramic understanding of the phenomenon. They are as follows:

➢ Identification of the main topics addressed in articles written on adolescent online and offline deviance until 2021;
➢ Comparing the psychological and sociological approaches on deviance and revealing overlaps;
➢ Highlighting the main predictors and indicators of online and offline deviance;

By systematically and qualitatively reviewing the literature in the field of deviance, we ultimately seek to gather the relevant findings on this field in a single paper and provide a better understanding of the same.

## Methods

As the main method, the article employs the 15 steps of systematic quantitative literature review proposed by Pickering and Byrne (2014). We began by defining the topic, formulating research questions, and identifying the keywords. Then, we searched and analyzed the relevant articles. At the same time, we followed the guidelines of PRISMA Protocol (Moher et al., 2009; Page et al., 2021) for ensuring that articles are systematically and transparently reviewed.

For the comprehensive analysis carried out in this study, we implemented computational text analysis methods using KH Coder (Higuchi, 2016), which allowed us to explore almost 500 articles. We employed this software for identifying patterns in the data and comparing different datasets by constructing a coding schema, which may be further used for analyzing other datasets. The use of a reproducible review technique, followed in the present article, has the major benefit that it diminishes researchers' subjectivity in conducting a review.

### Inclusion and Exclusion Criteria

The present research attempts to examine a large dataset of articles on deviance and online deviance from the Web of Science database. The rationale behind choosing Web of Science was to include only objective peer-reviewed articles from specific fields. It also facilitates the selection of articles based on the field of study using its filtering options.

The inclusion criteria were:

– Articles indexed in Web of Science
– Studies about deviance
– Language: English

The exclusion criteria used in the selection process consisted of:

– Field: at least one of the three of them should be Sociology (Web of Science filter)
– Studies that have an abstract available on the Internet in English language
– For Dataset 1: Articles' impact: top cited filter and most cited for the full-text articles
– For Dataset 2: Articles that refer to adolescent deviance (TS 2) and online adolescent deviance (TS 3) – for detail see section Stage 1 – Preparing the datasets

Considering the objective of this paper, for the analysis, we chose to include only those sources that have Sociology as one of their three focus fields. We employed this criterion in order to systematically narrow the extensive dataset while keeping focused on the impact of deviance in society. The included sources are articles, reviews, and book chapters in English, with an abstract. Furthermore, the sources belonging to the dataset have as one of their keywords “deviance” or “online deviance.” In terms of timeframe, we included in our systematic review the articles that were published between January 1975 and April 2021.

For objectively selecting the articles with the highest impact, we applied the highly cited articles filter on the previous results. Parallel to this dataset, we created a second dataset that included articles, reviews, and book chapters that also contain “deviance” and “adolescence” or “online deviance” and “adolescence” as their key focuses. In addition, all the selected sources were in English and had an abstract that was available for free.

The inclusion criteria were established to ensure the objectivity of sources selection and study replicability. As the main goal of our study was to offer an overview of the phenomenon of adolescent deviance, we did not use an extensive number of exclusion criteria.

### Computational Text-Analysis Using KH-Coder

Based on the analysis model proposed by Pickering and Byrne (2014), our systematic analysis, which uses KH Coder, followed three main steps:

➢ Stage 1 – Preparing the datasets (includes steps 1 to 6 according to Pickering and Byrne, 2014)
➢ Stage 2 – Identifying the coding schema (includes steps 7 and 8 according to Pickering and Byrne, 2014)
➢ Stage 3 – Analyzing the bulk set of articles (includes steps 9 to 11 according to Pickering and Byrne, 2014)
➢ Stage 4 – Presenting the main results (includes steps 12 to 15 according to Pickering and Byrne, 2014).

#### Stage 1 – Preparing the Datasets

Based on the above-mentioned terms, first, we made repetitive composed queries on the Web of Science database. For building our database, we chose words that referred to deviance in general, deviance as occurring in adolescence, and online deviance during teenage years.

The dataset was created by employing three main criteria (TS = Topic/Subject):

TS1 = (devian^*^ OR disruptive behavior OR immoral OR amoral OR harming practices OR incivility OR bad behavior OR harming behavior OR deviant behavior OR moral panic OR delinquency OR anomie OR immorality OR social disorder OR antisocial behavior);
TS2 = (devian^*^ AND adolescen^*^) OR TS = (devian^*^ AND teenage^*^) OR TS = (devian^*^ AND school^*^);
TS3 = (Online devian^*^ AND adolesc^*^) OR TS = (Online devian^*^ AND teenage^*^) OR TS = (Cyber^*^ AND adolesc^*^) OR TS = (Cyber^*^ AND teenage) OR TS = (Digital^*^ AND adolesc^*^) OR TS = (Digital^*^ AND teenage^*^);

From the obtained results, we constructed two datasets, one with the most cited articles (full text based on all three criteria) and a larger one comprising only abstracts (focused on adolescent deviance – criteria 2 and 3).

##### Preparing Dataset 1

Based on the mentioned queries, we selected 10% of the most cited articles from the Web of Science database by filtering the obtained results on the field. In this step, we took into consideration the top-cited articles on deviance related to the field of sociology. After applying this filter for the first criterion, 21 sources were found.

For the second criterion, three articles belonging to the highly cited articles were identified, while for the third criterion, only one article could be found. The four articles were already included from the previous stage. For completing the database with relevant articles in the field of teenagers' engagement in deviant behavior, we decided to include the 21 most cited articles that correspond to these two criteria such that the number of articles is the same as that for the first criterion. Further, we removed the duplicates and were left with 61 articles.

##### Preparing Dataset 2

This dataset comprises abstracts focusing on deviance and online deviance in the teenage years as found in the articles complying with the second and third criteria. In this step, we introduced 488 abstracts (from the 515 sources initially selected, we excluded 27 as they were duplicates or had no abstract).

#### Stage 2 – Identifying the Coding Schema

The abstracts of 61 articles were used for developing the coding schema, an extensive process that involves the following steps:

➢ Employing the preliminary word frequency, the automatic process using KH Coder software for identifying the most frequent terms;
➢ Manually organizing the most frequent words identified in the first step in thematic codes;
➢ Automatically generating self-organizing maps of words by automatically classifying them through Jaccard similarity coefficient using a multiple iteration process;
➢ Automatically generating self-organizing maps of our thematic codes and comparing them with the previously obtained automatic codes;
➢ Developing the coding schema by adding new codes based on the literature; at this stage, we manually assigned the terms of the 61 abstracts to the existing codes and created new ones.

In the end, we obtained 33 codes. The coding schema was furtherly employed for computational text analysis.

#### Stage 3 – Analyzing the Complete Dataset

Using the coding schema, we analyzed the full text of the most cited sources (61 articles) using the KH Coder software (through correspondence analysis).

Next, we examined the results of applying the coding schema to a new dataset, as well as the 488 abstracts on deviance and online deviance.

Before effectively applying the coding schema, we conducted a preliminary analysis. This includes making automated queries focused on terms frequencies and manual analysis of the 488 sources. The manual exploration facilitated a comprehensive understanding, thereby facilitating the acquisition of an overview of the field covered, the type of analysis, and the theories addressed, as well as additional aspects that could not be captured with the KH Coder software.

## Results

### Stage 1 – Preparing the Dataset

At this stage, we had 250,825 articles, out of which 2,874 are clustered in potentially relevant fields (e.g., sociology AND psychology; sociology; sociology AND criminology AND penology; sociology AND social issues).

For the second criteria, referring to adolescent deviance, we obtained a total of 3,800 sources and 374 results in the same potentially relevant fields.

For the third criterion, we obtained a total of 6,221 sources and 131 results in the same potentially relevant fields. The third criterion was more specific and included articles on online adolescent deviance.

**Figure 1:**
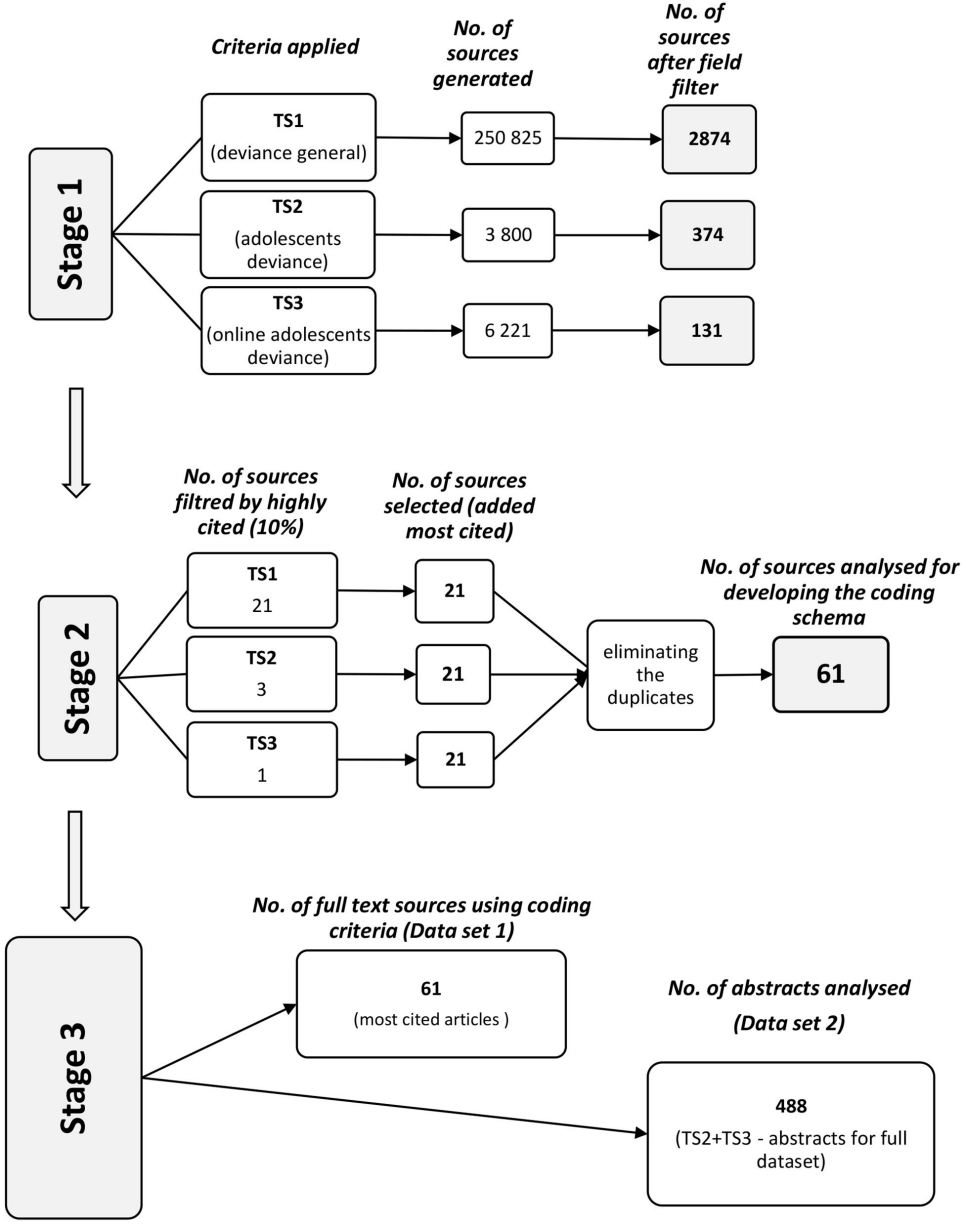
The systematic literature review process.

To facilitate an understanding of the process, we present the outline of the methodological approach below (Figure 1). The robustness of the schema was ensured by developing it based on PRISMA Protocol (Page et al., 2021); the employed PRISMA guidelines for Dataset 1 and Dataset 2 may be consulted in the Supplementary Material section.

### Stage 2 – Identifying the Coding Schema

The creation of the coding schema started by making queries to explore the relationships between words and their co-occurrences patterns. Then, we classified relevant terms into categories.

In the first phase of the analysis, we identified the most frequent words from the 61 abstracts. The list of the most common words includes “adolescent,” “health,” “social,” “online,” “school,” “family,” etc.

From the word association query, we defined the thematic categories.

Consequently, the word association revealed that there are seven major themes.

Next, we presented the main themes identified in the previous step and the frequency of the terms. With reference to the occurring medium, we distinguished the terms indicating the digital environment, namely, *online* (45), *digital* (41), *Internet* (39), *technology* (15), *Facebook* (10), *ICT* (9), *computer* (7), *informational* (6), *cyber* (5), and *cyberspace* (2), and the offline context: *family* (52), *school* (47), *life* (39), *parent* (24), *neighborhood* (14), *physical* (13), *offline* (110), *country* (5), and *gentrification* (7).

One interesting finding concerns the frequency of the *health* term (59), which seems, at first sight only, indirectly connected to deviance. The associated terms in this category include *mental* (18), *stress* (9), *autism* (7), *autistic* (6), *anxiety* (5), *medical* (5), *stressor* (5), *disorder* (4), *harm* (4), *psychological* (4), *hospital* (3), and *fitness* (3). Their occurrence may be the result of including a more general query in the selection criteria, which resulted in a high number of results (2,874 articles in sociology), even after applying the field filter.

The word frequency query also revealed the population of interest in the selected studies, namely, *adolescent* (71), *child* (31), *youth* (30), *student* (14), *undergraduate* (13), *teenage* (9), *teenager* (8), *adolescence* (5).

Moreover, the abstracts included in the dataset refer to specific behaviors perceived as deviant in the online and offline context. Hence, terms such as *cyberbullying* (10), *sexting* [sext (5), sexting (4), sexter (1)], suggest deviant behaviors online, while words such as *bullying* (12), *harassment* (10), *violence* (9), *aggression* (5), *violent* (4), *grooming* (2) indicate deviant offline behaviors. Along with specific deviant behaviors, the articles refer to deviance in general, comprising terms such as *deviance* (23), *deviant* (16), and *deviancy* (2). The way terms are employed changes if they are associated with the words *digital, online*, or *cyber*.

The last category revealed that most frequent words consist of terms reflecting delinquency, namely, *delinquency* (25), *delinquent* (7), *crime* (17), *criminal* (14), *arrest* (7), *incarceration* (9), *imprisonment* (5), *desistance* (4), *criminological* (3), and *illicit* (3).

**Figure 2:**
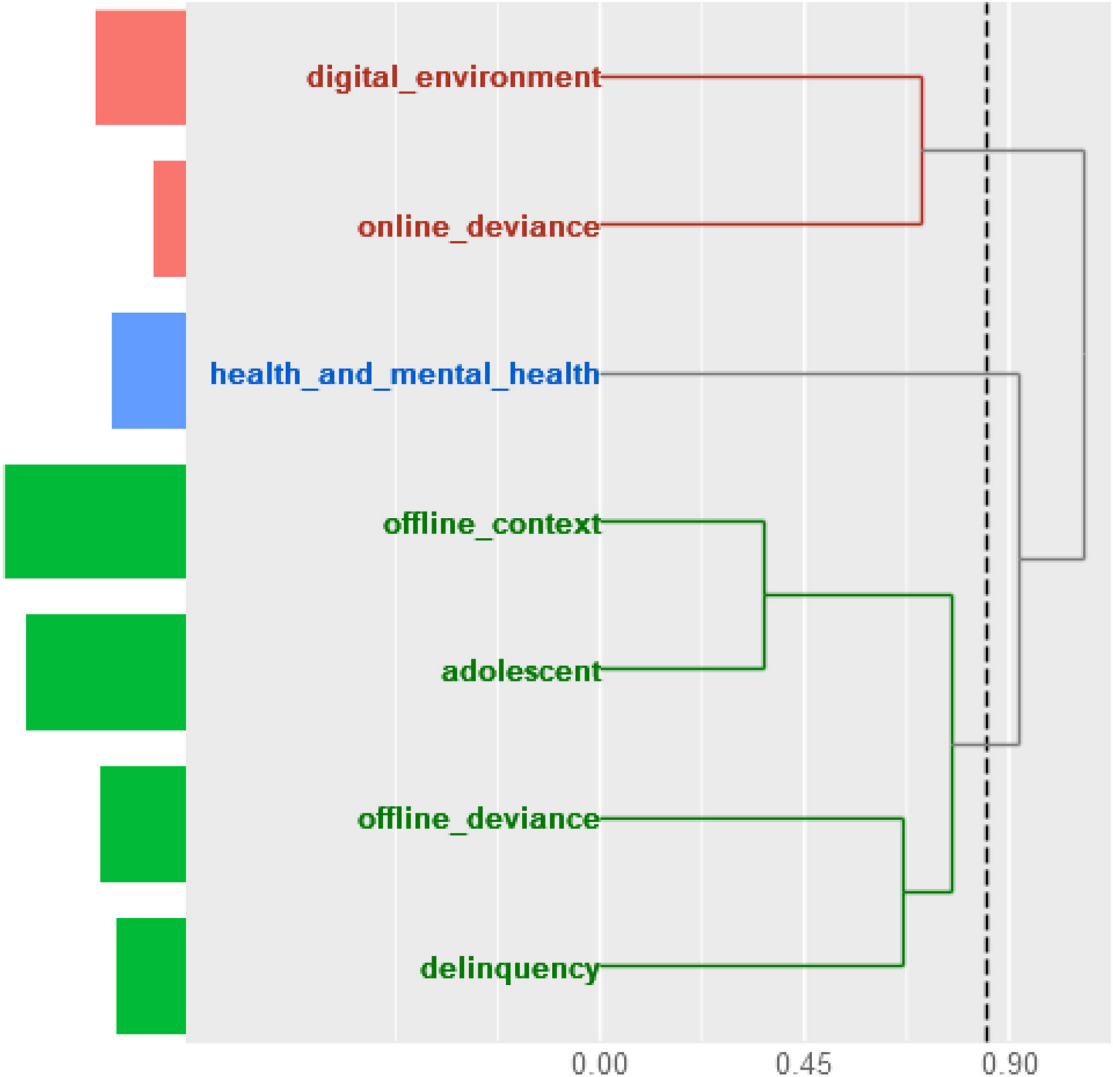
Hierarchical cluster analysis of the preliminary dimensions.

At this stage, we compressed the dimensions into three main clusters by employing hierarchical cluster analysis with Jaccard distance, as shown in Figure 2.

The first cluster included the terms related to the digital environment and online deviance. In the case of the second cluster, this included only terms related to health and mental health. The third one comprised the other codes, namely, offline context, adolescent, adolescent, offline deviance, and delinquency.

#### Improving the Initial Coding Schema

**Figure 3:**
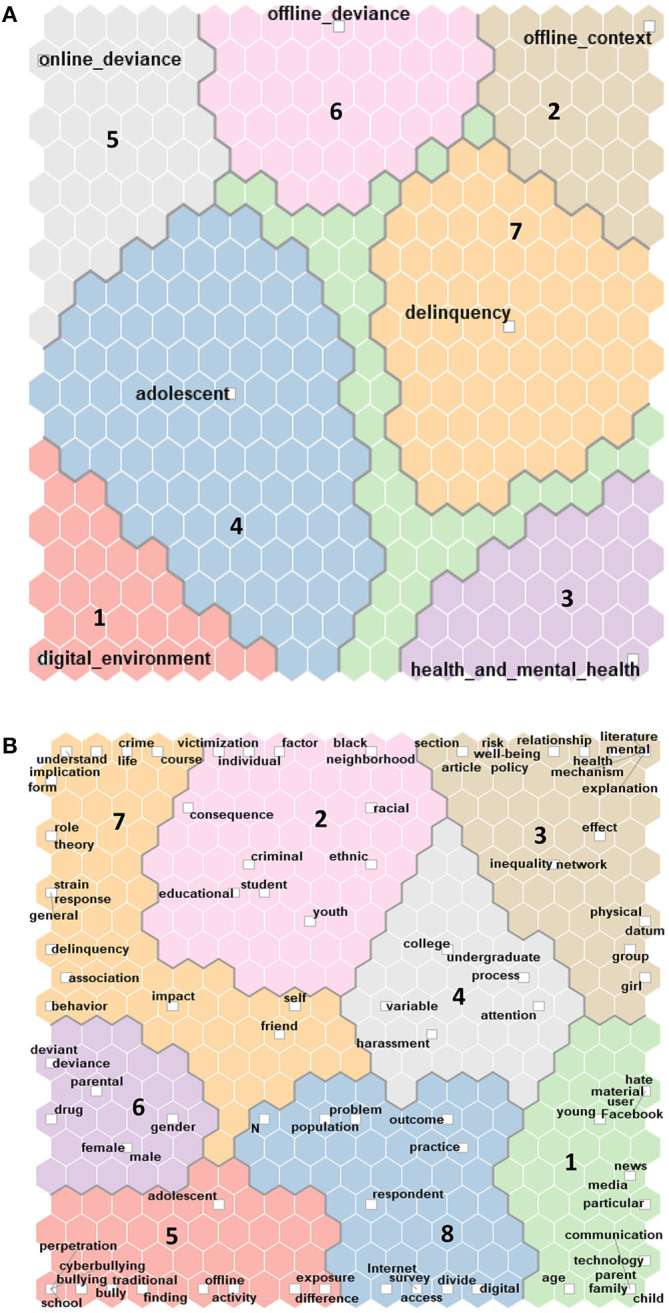
**(A)** Self-organizing map data set 1. **(B)** Self-organizing map data set 1.

For assessing the reliability of the created coding schema, we prepared a self-organizing map of the words from the abstracts the created coding schema, we prepared a self-organizing map of the words from the abstracts (Figure 3A) and compared it with the self-organizing map of the preliminary codes (Figure 3B).

The self-organizing map of abstracts partially reflected the map of codes that we developed, even if the employed colors did not coincide.

In the main clusters of the self-organizing map of abstracts, we identified key terms that relate to the distinguished dimensions (a. digital environment; b. offline context; 3. health and mental health, d. adolescent; 5. online deviance; 6. offline deviance; 7. delinquency). The other emerged category consists mostly of terms related to divide and inequality, including the issue of digital divide and differential access to resources (8). The least consistent category between those created using word frequency query is related to *adolescence*. Considering the finding of comparing the two figures generated from preliminary coding and self-organizing map of words, we reviewed the classification by manually coding each abstract.

#### The Final Coding Schema

**Figure 4:**
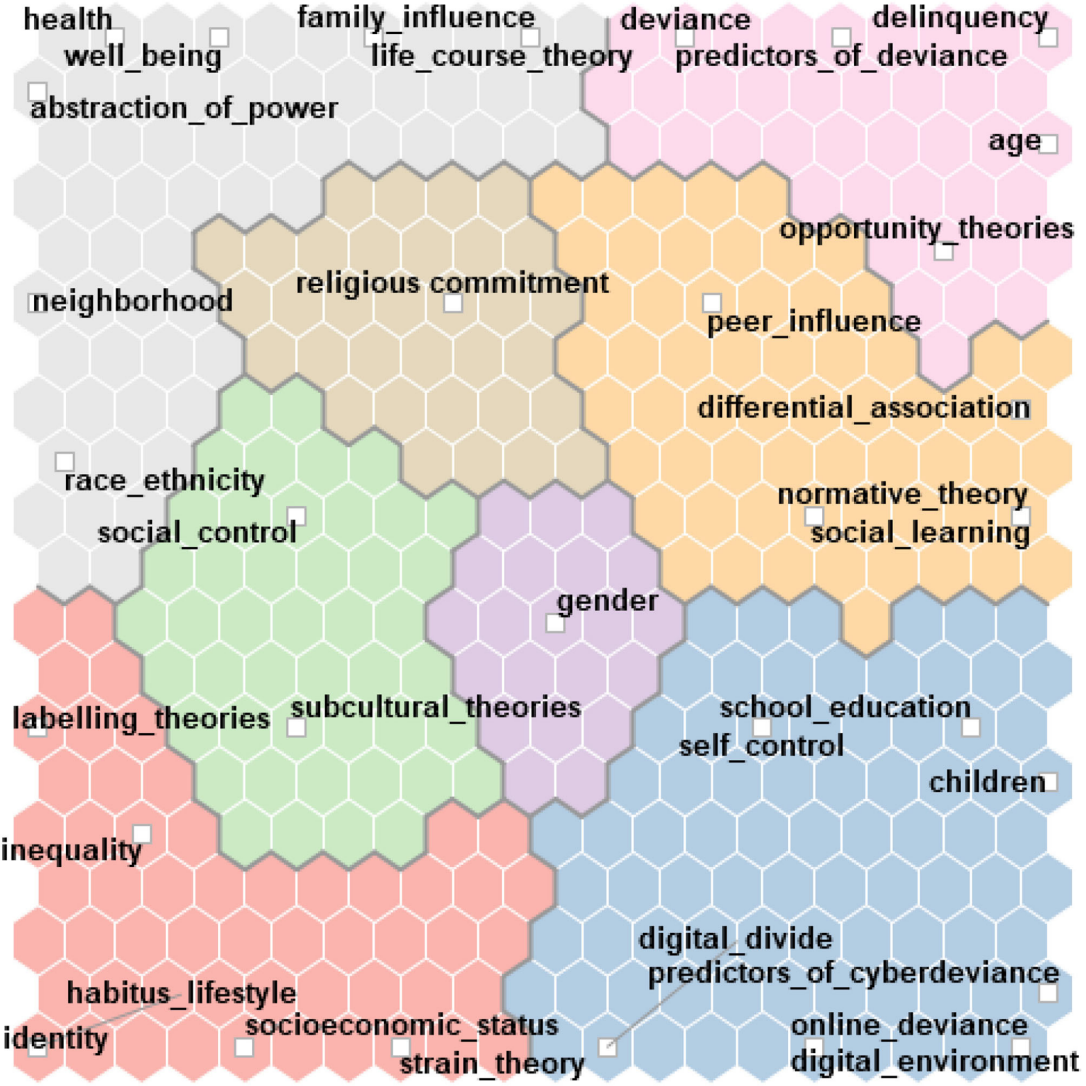
The coding schema.

At this stage, we added specific codes to the preliminary classification for including the predictors of deviance, as well as of cyber-deviance mentioned in the abstracts and codes that relate to the main influencing factors. In the end, the final coding schema had 33 codes, with the most recurrent being predictors of deviance, school and education, socioeconomic status, age, children and adolescents, discipline and power relations, family factors (Figure 4). The final codes were grouped into eight clusters using a self-organizing map with the Jaccard similarity coefficient query.

Along with the substantive categories mentioned above, we identified terms referring to the main theoretical frameworks such as *social learning, opportunity routine theories, differential association, normative theory, self-control, social control, strain theory, subcultural theories, life course theory, digital divide*, and *labeling theories*. Moreover, we created specific codes for terms related to lifestyle (habitus lifestyle), health, and well-being.

**Figure 5:**
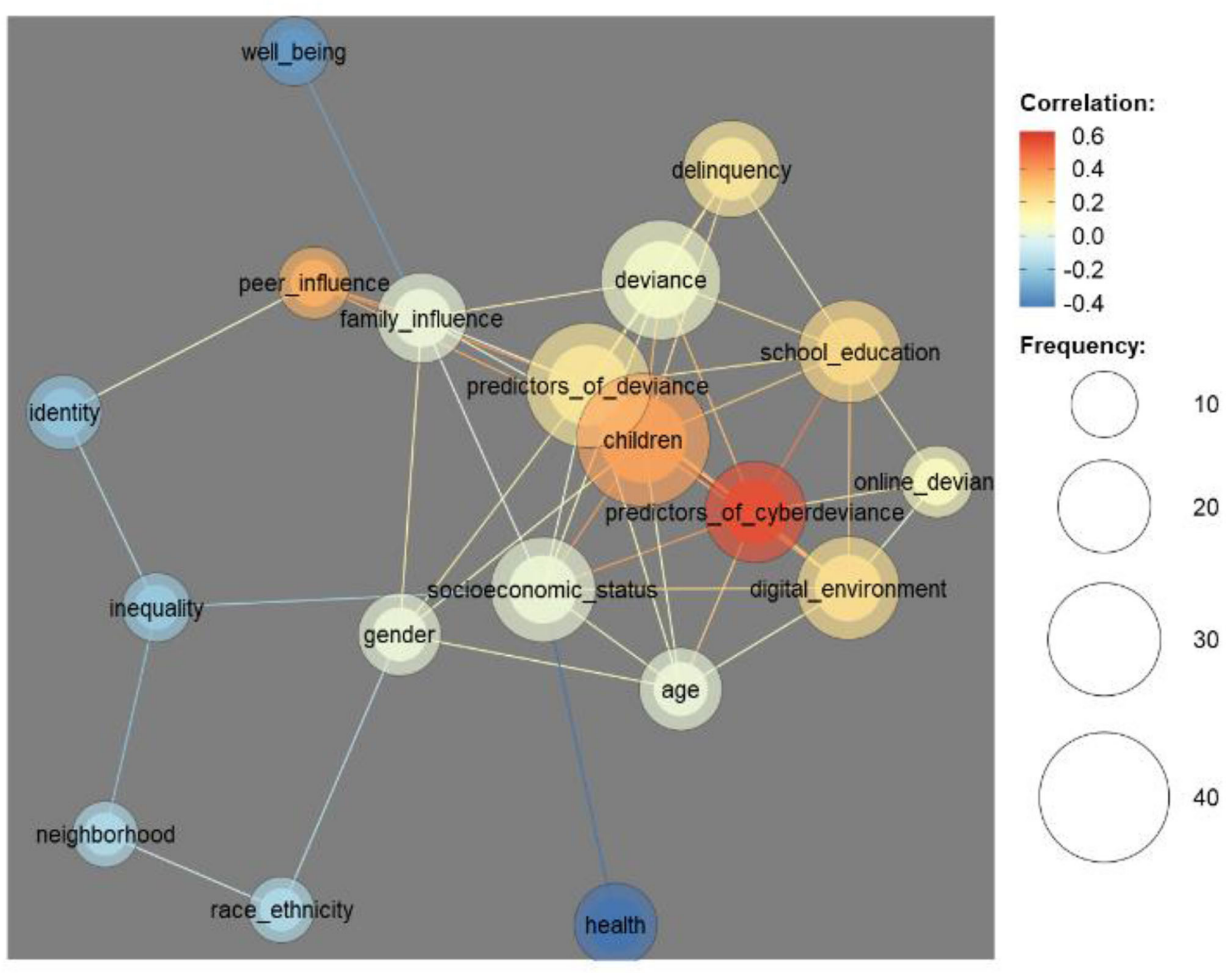
Co-occurrence network of the most encountered codes as applied to abstracts.

For a better understanding of the resulting clusters, we generated a co-occurrence network of codes, which is based on the probability of two codes appearing in the same abstract. The network revealed correlations between the predictors of cyber-deviance, deviance, peer influence, children, school and education, delinquency, and deviance and digital environment (Figure 5). At the same time, terms related to health, well-being, and neighborhood are negatively correlated to the items mentioned above. Figure 5 also reveals the connections among identity, inequality, neighborhood, and ethnicity codes.

After inspecting the correlations among codes, we considered the coding schema as final and employed it for the following analysis procedures.

### Stage 3 – Analyzing the Complete Data Sets

Stage 3 consisted of applying the coding schema to the two established datasets (61 full text and 488 abstracts) and compared the results with the hierarchical word clusters through correspondence analysis and crosstabulations.

#### Dataset 1 (61 in Extenso Articles)

The analysis of the first dataset was conducted in four stages, consisting of correspondence analysis and crosstabulations of hierarchical clusters and the coding schema (unit of analysis: articles). The third stage consisted of examining the associations among 33 generated codes using a similarity matrix to identify overlaps. The last procedure involved creating a detailed network between predictors of interest in the field of deviance and online deviance.

**Figure 6:**
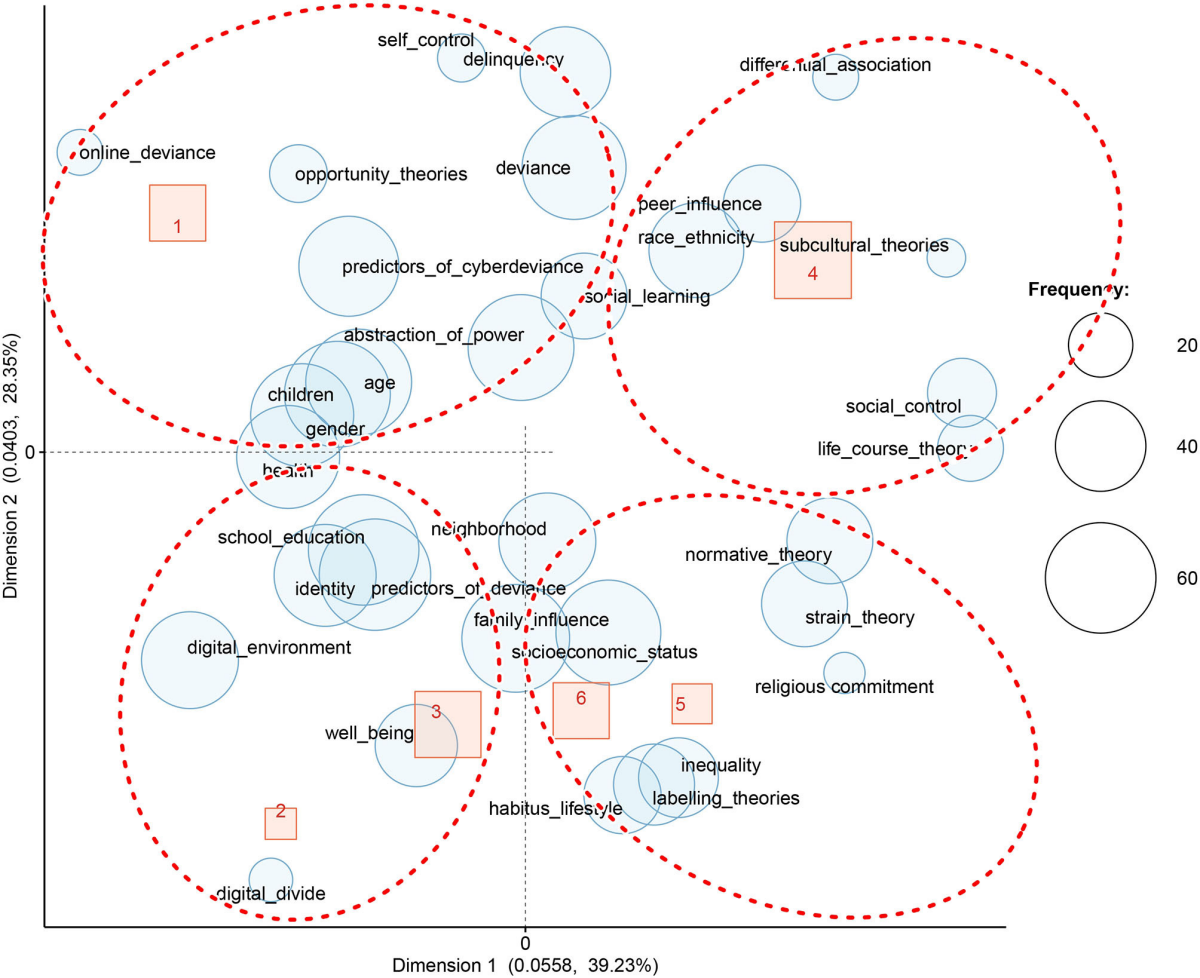
Correspondence analysis of codes and hierarchical clusters in extenso articles.

*The correspondence analysis* revealed co-occurrent topics. This method revealed four *latent groups of topics*, namely, online deviance, identity, and communication in the online sphere, peer influence, and explanatory theories of deviance (See Figure 6 below). The first identified cluster mostly overlaps with the nodes related to online deviance, predictors of cyber-deviance, predictors of deviance, delinquency, and opportunity theories. At the periphery of this class, we found terms that refer to children, gender, age, and health. In total, the first *group of topics* includes 10 articles. The second *group*, which encompasses cluster 2 (3 articles) and 3 (14 articles), focuses on aspects related to identity and communication in the online sphere. The theory of the digital divide and the articles referring to well-being are also included in this group. The fourth cluster forms the *third group* on its own, and it includes 19 articles. For this cluster, the most representative codes are peer influence, ethnicity, differential association theory, and the subcultural approach. The *last group* is composed of clusters 5 (5 articles) and 6 (10 articles), and it presents an integrative view. Thus, as the main codes may show, articles that question the causes of deviance (social control, life-course theory, normative theory, and strain theory) and articles that focus on understanding deviance as a social construct (labeling theory, the relationship between lifestyle, deviance, and social inequality) belong to this category.

**Figure 7:**
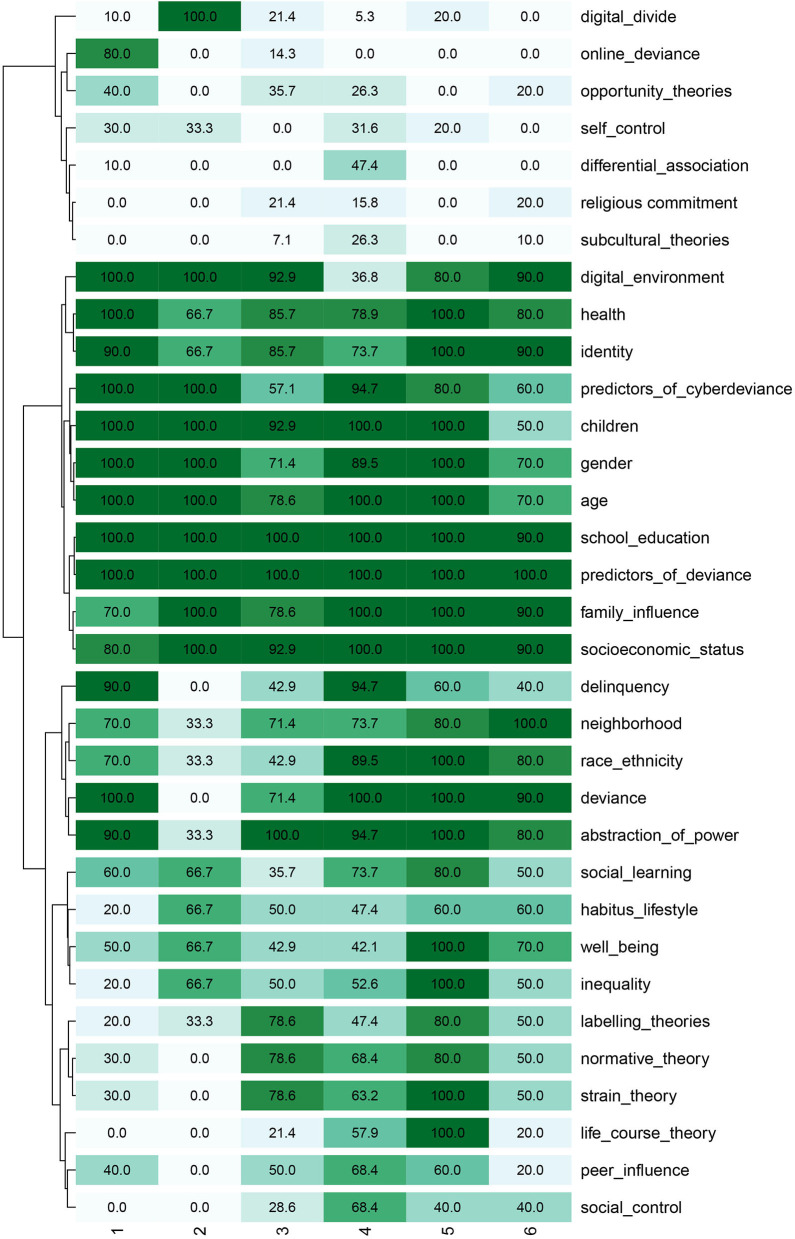
Heat map of the in extenso articles.

A more in-depth examination of the clusters is possible with the use of the crosstabulations available in KH Coder. The results of this examination allowed the identification of the most encountered codes and their statistical signification in establishing the clusters. Thus, the most frequent themes covered in the articles are predictors of deviance (61 articles), school education (60 articles), socioeconomic status (57 articles), age (55 articles), children& adolescents (55 articles^**^^1^ – present in clusters 1, 2, 3, 4, and 5), abstraction of power (55 articles^*^ – present in clusters 1, 3, 4, 5, and 6), family influence (54 articles), deviance (53 articles^**^ – present in clusters 1, 3, 4, 5, and 6), health (52 articles), gender (52 articles), and identity (51 articles), as presented in Figure 7.

The other codes that influenced the composition of the clusters are digital environment (46 articles^**^ – present in clusters 1, 2, 3, 5, and 6), online deviance (10 articles^**^ – present mostly in cluster 1), delinquency (40 articles^**^ – present mostly in clusters 1 and 4, and to a lesser extent in cluster 3, 5, and 6), differential association (10^**^ - present mostly in cluster 4 in 9 articles and in 1 article from cluster 1), social control (23^**^ - present mostly in cluster 4 in 13 articles and in clusters 3, 5 and 6), life-course theory (21^**^ – present mostly in clusters 4 and 5 and to a less extent in clusters 3 and 6), digital divide (9^**^ – present mostly in cluster 2 and to a less extent in clusters 1, 3, 4, and 5), normative theory (36^*^ – present in clusters 3, 4, 5, and 6) and strain theory (36^*^ – present mostly in clusters 3, 4, and 5 and to a less extent in cluster 1).

The similarity matrix of codes shows consistent overlaps among the nodes included in the coding schema. Thus, terms belonging to the codes, namely, predictors of cyber-deviance, deviance and predictors of deviance, family influence, school and education, gender, age, socioeconomic status, children, identity, abstraction of power, and delinquency, have a correlation value higher than 0.5. The most distinctive nodes have fewer occurrences, including online deviance, religious commitment, opportunity theories, differential association, self-control, subcultural theories, digital divide, life-course theory, and social control.

Further, we generated two networks, including the predictors of deviance and online deviance, for a more in-depth inquiry. The resulting networks facilitate the identification of the terms that are mainly associated with them.

As revealed by the presented network, the most encountered predictors for deviance are paternal incarceration, violent victimization, socialization, selection, parental deviance, socioeconomic status, family factors, parental control, parenting practices and monitoring, school factors, self-efficacy, peer affiliation, popularity, network friendship, self-control, alcohol and substance use, educational attainment, deviant exposure, delinquent parents, aggression, neighborhood disadvantage, risk, and others.

**Figure 8:**
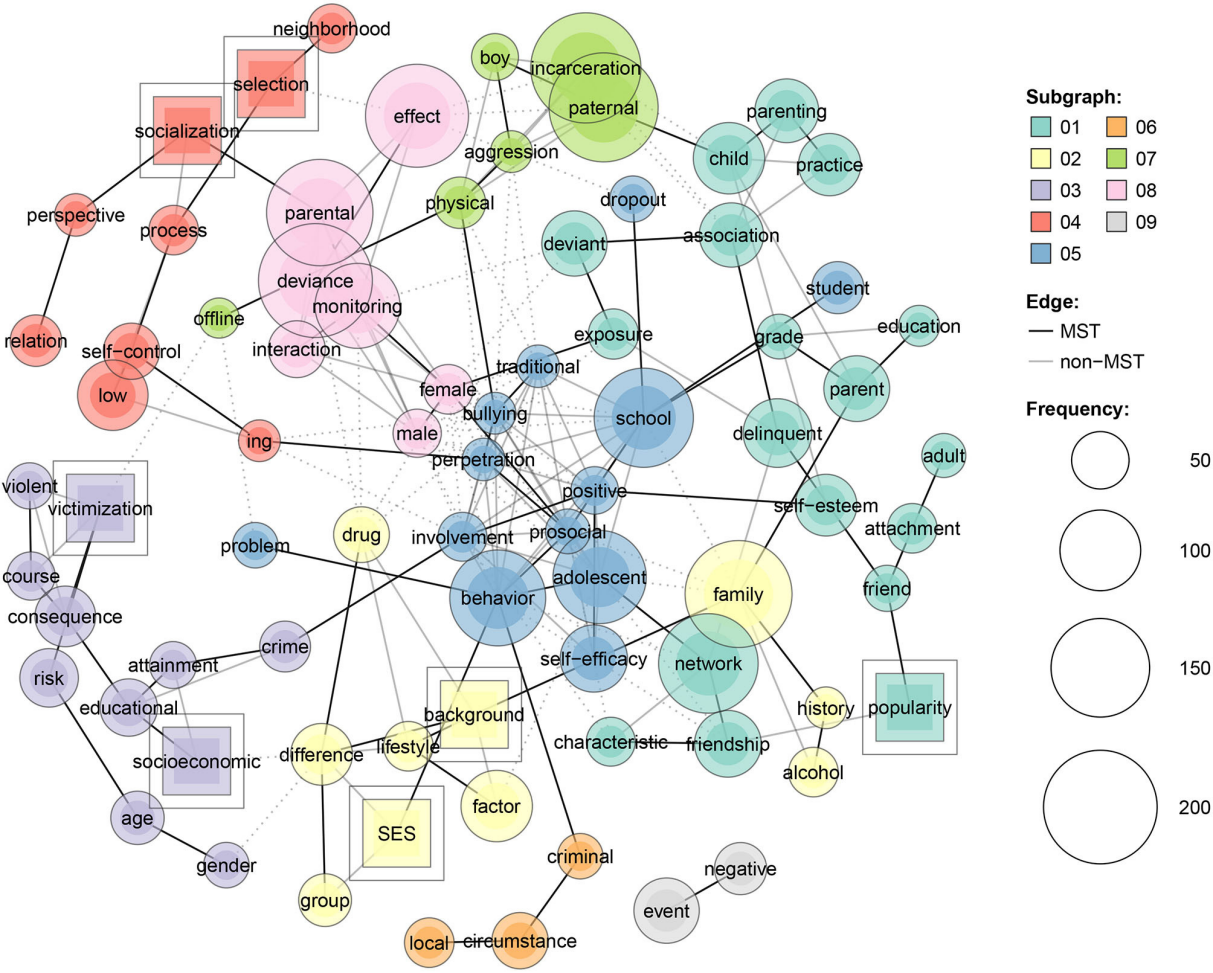
Predictors of deviance grounded on modularity procedure.

Given the grouping of predictors based on technical modularity shown in Figure 8, we identified five main categories of predictors of deviance: family patterns (parental deviance, parental monitoring, paternal incarceration, parenting practices, etc.), socio-demographic aspects (SES difference, life style background and drug abuse, family background, etc.), socialization (low self-control, socialization process, selection process, etc.), victimization (violent victimization, risk, age and gender risk, life-course consequences, etc.) and school and individual factors (traditional bullying, school grades, adolescent self-efficacy, school drop-out, etc.).

**Figure 9:**
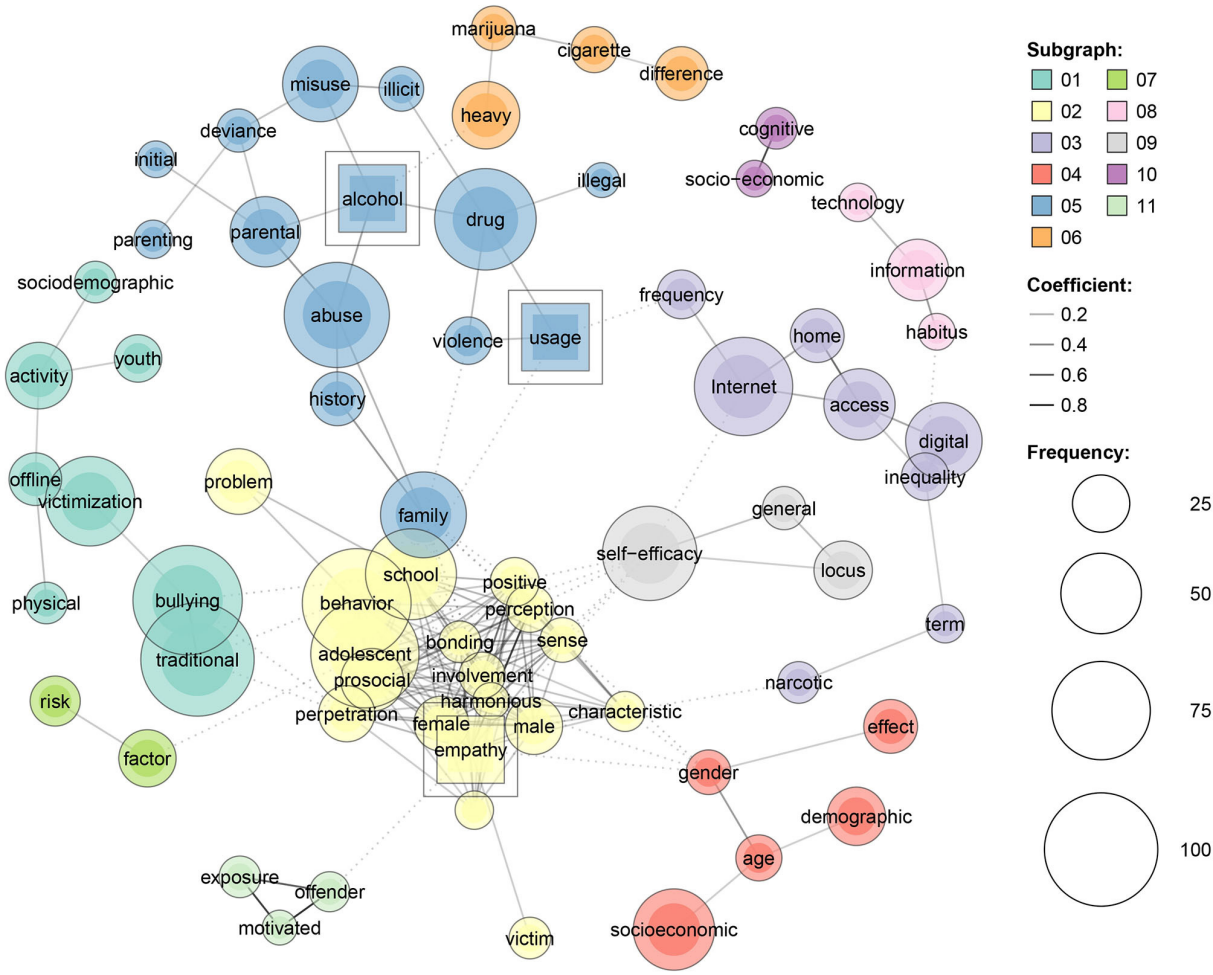
Predictors of cyber deviance grounded on modularity procedure.

In relation to cyber-deviance (see Figure 9), the frequent predictors for cyber deviance encompass the following: traditional bullying, offline victimization, parental abuse, alcohol and substance use, Internet access, Internet misuse, compulsive Internet use, substance use, online exposure, parental control, school factors, self-efficacy, socioeconomic status, parenting, digital inequality, information habitus, etc.

With regards to the most common predictors of online deviance, they can be grouped into five categories: family patterns (parental abuse, parental deviance, family history and abuse, parenting style, etc.), socio-demographic aspects (gender and age effects, demographic and socio-economic, etc.), victimization (offline victimization, traditional bullying, physical and offline activities, exposure, etc.), school and individual factors (school problems, school bonding, school involvement, empathy, school perception, school behavior, prosocial involvement, etc.) and Internet and computer use (home Internet access, digital inequality, frequency usage of Internet, technology information habitus, etc.).

#### Dataset 2 (488 Abstracts)

The last stage of analysis recommended by Pickering and Byrne (2014) consists of adding the whole set of articles that correspond to the searching criteria.

The exploration of the second dataset required conducting preliminary analysis, considering its extensive dimension. Consequently, we added two additional stages, a manual assessment of the study field of research methods and a frequency query. The analysis of the second dataset was conducted in five stages: manual inspection of study field and research methods, word frequency, correspondence analysis of clusters and codes, crosstabulations of clusters and codes, and similarity matrix.

As mentioned already, the preliminary review was a manual analysis of 488 articles to gain an overview of the types of the articles. Note that, our first criterion of selection was the field of sociology. Thus, all 488 articles were from the field of sociology. Along with sociology, 143 articles were also from the field of psychology, 131 from the field of criminology, and 117 published in other fields such as education, communication, religion, health, biomedical, social sciences, etc.

Of the 488 sources analyzed, only a few (**2**) were systematic analyses; **16** were literature reviews; **16** were aimed at reviewing theories or theoretical issues in general; **348** used quantitative methods, **93** qualitative methods, and **13** a mixed approach (quantitative as well as qualitative methods).

From the complete dataset, the articles mostly approached strain theories, closely followed by social learning, self-control, and social control theories. Opportunities and routine theories, bonding theory, differential association theories, reinforcement theory, life-course perspectives, and social disorganization theory are other main perspectives.

**Figure 10:**
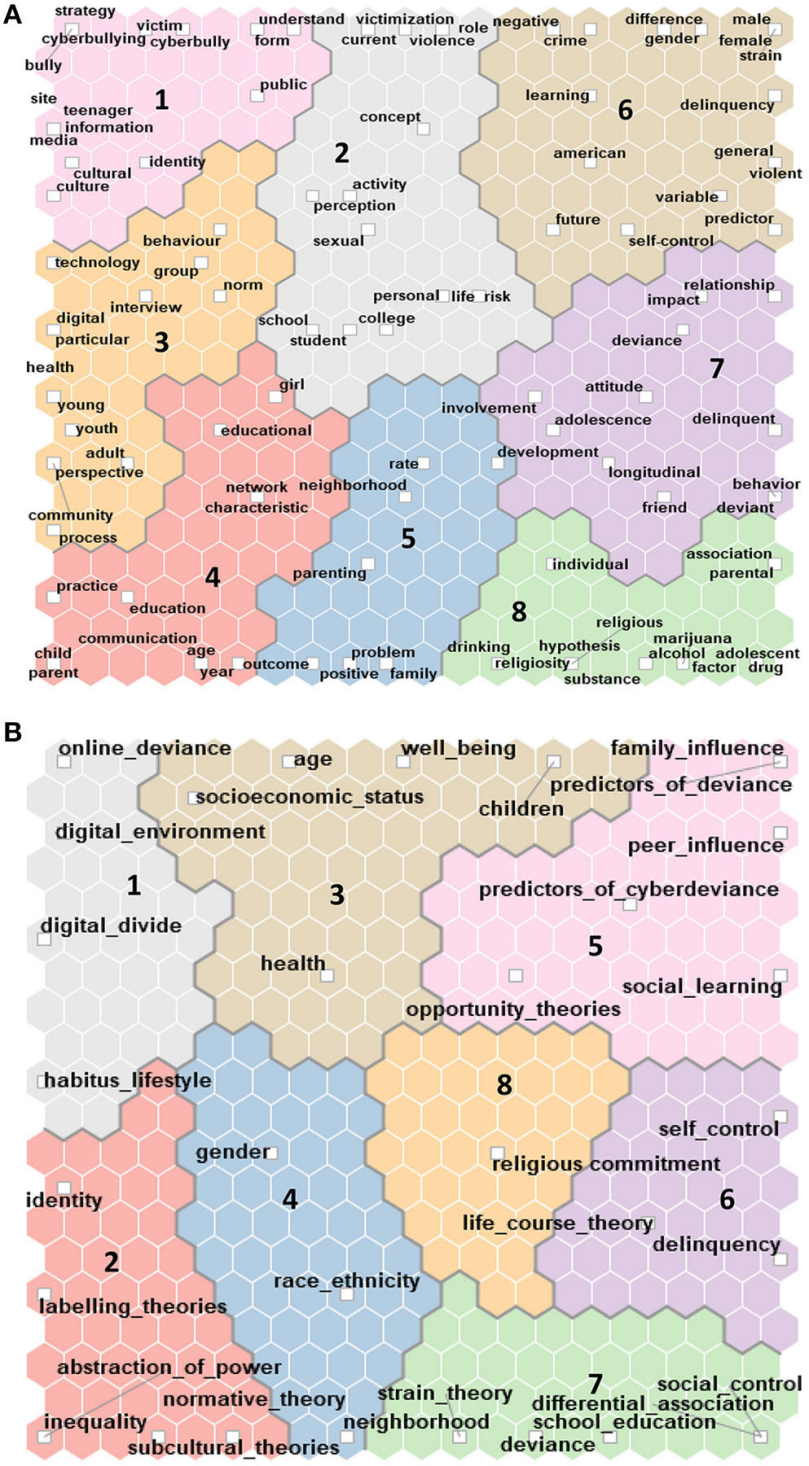
**(A)** Self-organizing map Data set 2. **(B)** Self-organizing map Data set 2.

The next stage consisted of generating a self-map analysis based on the terms' frequency and cluster identification (Figures 10A,B)

After employing word frequency and self-map analysis for data exploration, we examined the network among hierarchical clusters generated using the Ward technique with the Jaccard coefficient and the topics defined in our coding schema.

The inclusion of a higher number of sources in dataset 2 created a clearer demarcation between the observed categories. The classification suggests a structure of four *groups of topics*, similar to those identified in the first dataset. The specificity of the current classification makes it better at distinguishing socio-constructivist theories and the predictors of deviance. Thus, the main groups consist of predictors of deviance and cyber deviance (clusters 1 and 2), online deviance and digital environment (clusters 3 and 6), post-positivist and integrative theories of deviance (cluster 5), and a smaller group related to a constructional approach of deviance focused on identity, inequality, and power relations (cluster 4).

**Figure 11:**
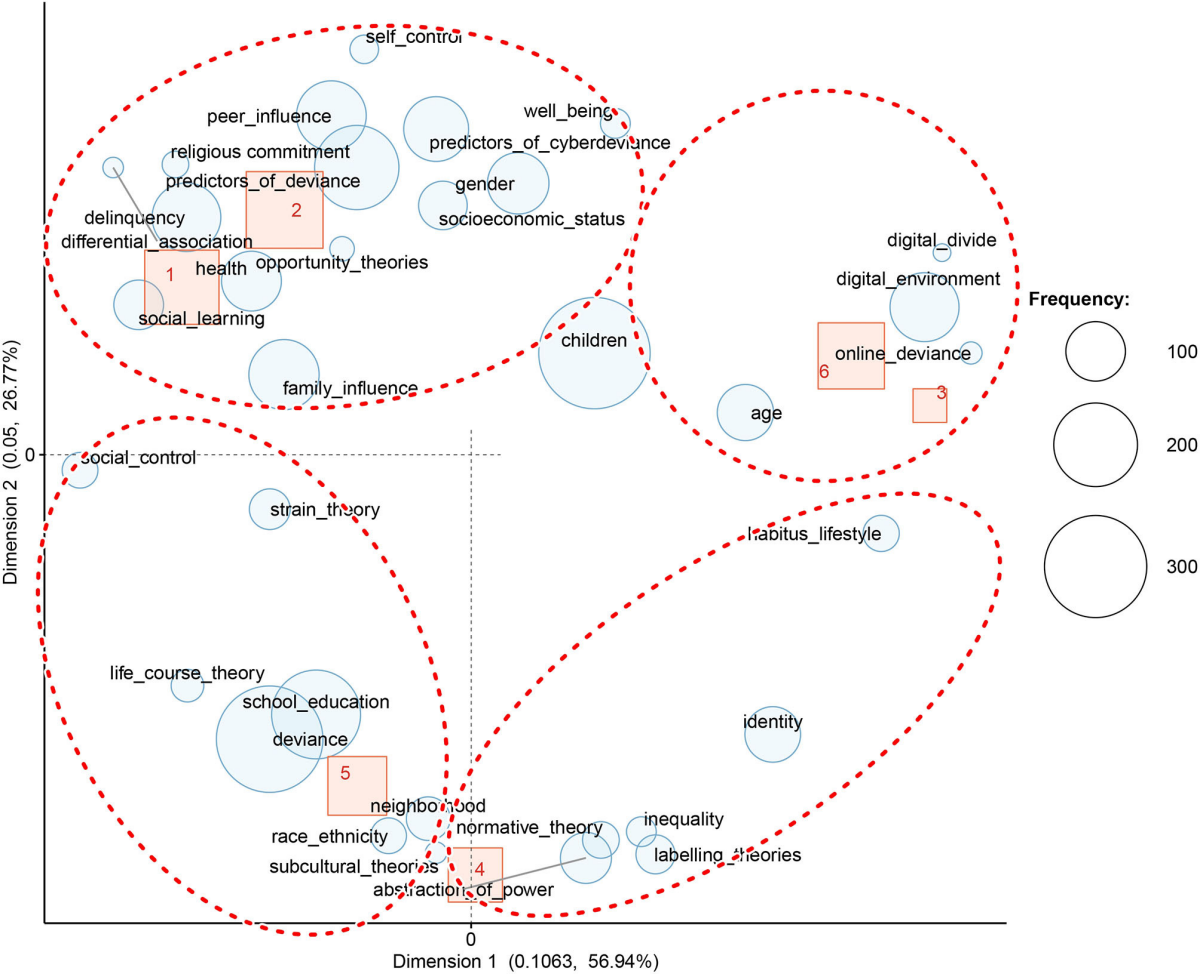
Correspondence analysis of codes as hierarchical clusters abstracts from the whole dataset.

As Figure 11 illustrates, the first category, composed mostly of cluster 1 (116 abstracts) and cluster 2 (124 abstracts) data covers the codes, namely, family influence, peer influence, school and education, predictors of cyber-deviance, predictors of deviance, strain theory, religious commitment, self-control, social control, gender, social learning, differential association, opportunity theories, health, well-being, and socioeconomic status.

A second category, which encompasses cluster 3 (23 abstracts) and cluster 6 (92 abstracts) data comprises terms related to online deviance, digital divide, digital environment, and age. It emphasizes aspects related to patterns of online communication and deviant behaviors in the cyber-environment.

The third class (72 abstracts of cluster 5) presents an integrative post-positivist approach to deviance and includes main theories such as social control, strain theory, life-course theory, subcultural theory, and social disorganization perspective (creating inequalities among neighborhoods as a cause of deviance). Considering that the main nodes of this class represent deviance and education, it may be assumed that this class investigates the relationship between deviance and education.

The last class presents a constructivist approach, including the following codes: labeling theories, inequality, identity, abstraction of power, habitus lifestyle, and normative theory (norms/normative theory). This class is weakly associated with the automatically constructed hierarchical clusters (a part of cluster 4 that is composed of 61 abstracts). As the figure shows, the categories of lifestyle and identity are located far from the main center, moving closer to class 2.

**Figure 12:**
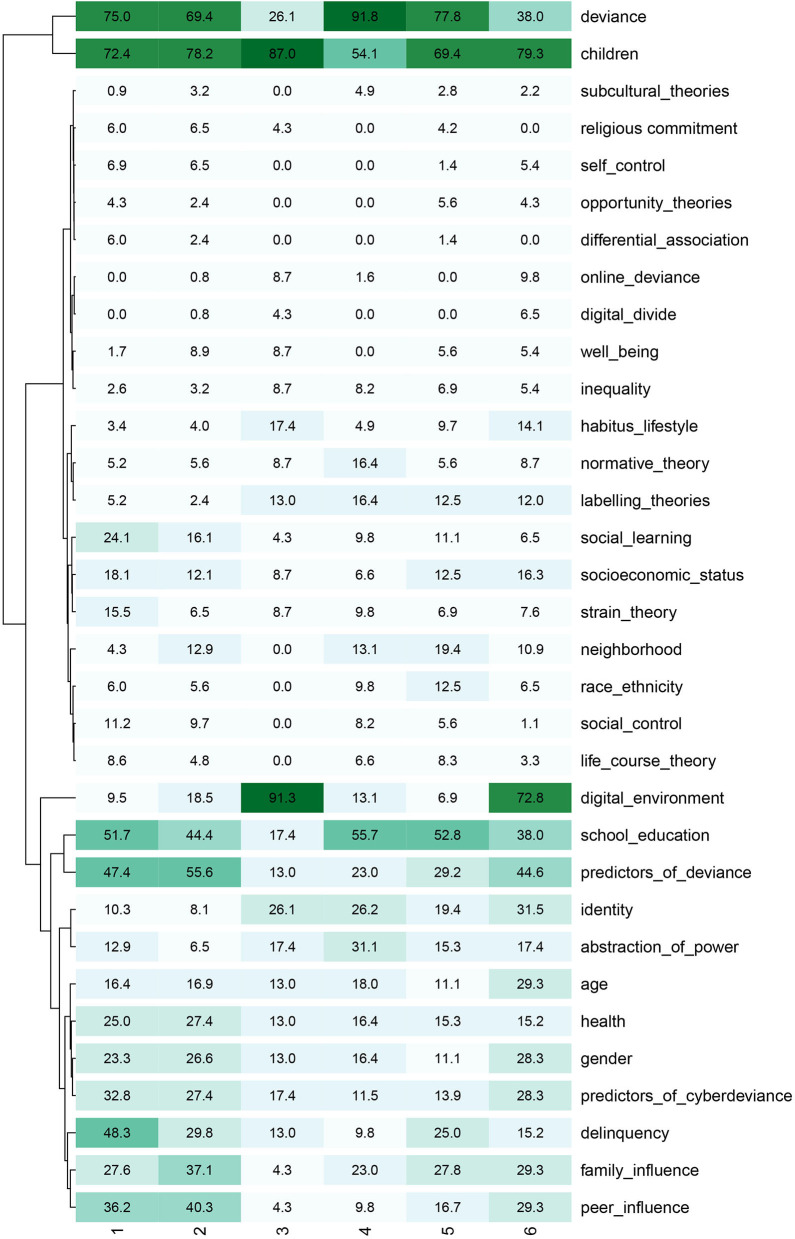
Crosstab bulk articles heatmap filtered.

We proceed with inspecting the associations among hierarchical clusters with crosstabs, which allow us to indicate the recurrent themes (Figure 12).

The most frequent nodes in the 488 abstracts are children and adolescents (357^**^ abstracts – present in all clusters, but in only a few abstracts in cluster 4), deviance (326^**^ – present mostly in clusters 1, 2, 4, and 5 and in a few abstracts from the other clusters), school education (226^**^ – present mostly in clusters 1, 2, 4, and 5), predictors of deviance (203^**^ – present mostly in clusters 1, 2, and 6), family influence (140^*^ – present in clusters 1, 2, 4, 5, and 6), peer influence (138^**^ – present in clusters 1, 2, 5, and 6), digital environment (135^**^ – present mostly in clusters 3 and 6 and to a less extent in clusters 1, 2, 4, and 5), delinquency (134^**^ – present mostly in clusters 1, 2, and 5 and to a less extent in clusters 3, 4, and 6), predictors of cyber-deviance (119^**^ – present mostly in clusters 1, 2, and 6 and to a less extent in clusters 3, 4, and 5), gender (107 – present in every cluster), and health (101 – present in every cluster). The structure of clusters is also significantly influenced by the following topics: online deviance (13^**^ mostly present in cluster 6), identity (87^**^ – mostly present in clusters 3, 4, and 6 and to a less extent in clusters 1, 2, and 5), social learning (69^**^ – mostly present in clusters 1 and 2), abstraction of power (73^**^ – mostly present in clusters 3, 4, and 6 and to a less extent in clusters 1, 2, and 5), digital divide (8^**^ – present in cluster 6), labeling theories (42^**^ – mostly present in clusters 4, 5, and 6), lifestyle (36^**^ – mostly present in clusters 3, 5 and 6 and to a less extent in clusters 1, 2, and 4), social control (35^*^ – mostly present in clusters 1 and 2), differential association (11^*^ – mostly present in cluster 1), neighborhood disadvantage (53^*^ – mostly present 2, 4, 5, and 6). While for the first dataset, the similarity matrix shows consistent overlaps among the codes included in the coding schema, the second dataset reveals consistent differences among the codes. Thus, the highest similarity values are between deviance – children and adolescents (0.535), children and adolescents – predictors of deviance (0.414), deviance – predictors of deviance (0.381), and peers influence – predictors of deviance (0.375).

### Predictors of Deviance and Cyber-Deviance in Social Sciences

Given the high concern of researchers for the predictors of deviance and online deviance, we have provided a more detailed review of them predictors. Therefore, the present review attempted to provide more clarity on the most encountered predictors of deviance. To provide an understanding and an overview of this field, we considered it necessary to categorize these predictors, which are widely presented in the literature. The analyses presented show that the most common predictors of deviance relate to family patterns, socio-demographic aspects, socialization, victimization, and school and individual factors. Family, peer group, and school are the main social spaces of adolescents. In addition, considering the impact of the Internet and Web 2.0 technologies on socialization, identity formation, and leisure, we explored the predictors of offline and online deviance.

#### Family Patterns

Family relationships have a major influence on teenagers' level of engagement in deviant acts by loosening social constraints - Loeber and Stouthamer-Loeber (1986), Thornberry (1987). Centered on the impact of parents' deviant behavior and attitudes on children's delinquency, deviant behaviors and attitudes paradigm analyzes family deviance, which includes family disorganization, parental involvement in lawbreaking acts and parental deviant values, and tolerating attitude related to dishonesty and criminality, as major risk factors of engagement in deviant conducts. According to the disruption paradigm, conflicts between parents, the absence of a parent, parent separation, and divorce affect children's conduct directly and indirectly. Variables such as inappropriate parenting practices and parental supervision (Sampson and Laub, 1992, 1994; Zhang and Messner, 1995; Wiesner and Shukla, 2018) and family structure aspects including broken home, household size, sibling rank, and family environment (LeFlore, 1988) are worthy to be taken into consideration while studying deviance in general and teenagers' deviance, in particular. The relationship between parents is considered a very important factor in adolescents' engagement in deviant acts. The better the parents' relationship and the more they provide a peaceful environment for their child to grow up in, the less likely they are to engage in deviant acts (Lu et al., 2020). Harsh parental discipline (Hong et al., 2017), depression, and school engagement are other risk factors of deviance in teenagers (Lin and Yi, 2016) related to family deviance and family functioning.

#### Socio-Demographic Aspects

Researchers indicate a link between living in a certain area of a city and deviance. Grounded on the seminal work of Wilson (2012) regarding the criminalization process of people living in poor neighborhoods, *the underclass*, sociologists have noticed that there is a high rate of crimes and delinquency in such disadvantaged neighborhoods, which are inhabited by jobless people. The link between deviancy and the neighborhood context represents a factor that mediates the link among peer influence, family context, and deviance (Sampson and Laub, 1997; Sampson et al., 2002; Hwang and Sampson, 2014; Billings and Hoekstra, 2019). In addition, the structural inequality mirrored by urban topology is amplified by network effects (DiMaggio and Garip, 2012). Recently, network research scientists also demonstrated that income inequality is correlated to network fragmentation, which implies that an increase in social and income inequality is visible when social network fragmentation interacts with neighborhood distribution (Tóth et al., 2021).

#### Socialization

Association with deviant peers is related to the engagement in deviant and delinquent behavior (Agnew, 1991; Warr and Stafford, 1991; Sutherland et al., 1992; Matsueda and Anderson, 1998; Haynie and Osgood, 2005; Akers, 2017). The inquiry of whether one is deviant because one belongs to a deviant group, encompassed in the *socialization theories* (Agnew, 1985, 1991; Warr and Stafford, 1991; Dishion and Tipsord, 2011; Lin and Yi, 2016; Akers, 2017; McGloin and Thomas, 2019; Schwartz et al., 2019), or whether one chooses to be a part of a group with antisocial behavior because of a personal inclination for deviance, known as *selection mechanism* (Matsueda and Anderson, 1998; Haynie and Osgood, 2005; Barnes et al., 2006; Schwartz et al., 2019; Gallupe et al., 2020), helps capture this causal relationship.

Contrary to the perception of social learning theorists that deviance is a result of socialization with peers that have antisocial behavior, social control theorists hold that getting involved in crimes is a process that does not require any learning (Hirschi, 1969; Hirschi and Gottfredson, 2000; Costello and Zozula, 2016). A slightly different approach is taken by routine activity theorists who hold that deviant friends provide more opportunities to engage in norm-breaking and law-breaking activities (Osgood et al., 1996; Haynie and Osgood, 2005; Boman et al., 2019; Hoeben et al., 2021). Considering Giordano's observations regarding the need to explain peer relations from a multidimensional perspective (Giordano, 2003), researchers assess the impact of conflict (Boman and Mowen, 2019) and friendship quality (Poulin et al., 1999; Boman et al., 2019) on deviant behaviors of individuals.

#### Victimization

Researchers (Gorman-Smith et al., 1998) have established that there are significant associations among the profiles of offenders and patterns of family involvement, with non-offenders being more likely to have families with minimal problems, with serious chronic offenders belonging to families with many problems, such as issues of neglect, and with escalating offenders having a conflictual family background that is characterized by disruption. In the case of escalating offenders, the influence of family functioning toward engaging in deviant acts seems to be corroborated with peer deviance, the authors conclude. Researchers proved that peer offending represents a more powerful predictor of cyber-deviance, in general, and cyberpiracy, in particular, than low self-control (Lee, 2018; Lee et al., 2018). In the case of cyberbullying, exposure to risky online content and cyberbullying victimization, along with gender, school grade, school control, and perception of cyberbullying factors have significant impacts (Bae, 2021).

#### School and Individual Factors

As Davies (1995, 1999) emphasizes, the socio-economic background is a weak predictor of deviance, while difficulties with school, operationalized as lower grades and the likeness to drop out of school, strongly predict engagement in deviant acts. Despite the way the causal relationship between academic failure and deviance is questioned (Phillips and Kelly, 1979), there is no doubt that school deviance correlates to academic underachievement. Researchers have found that socioeconomic status, cultural capital, and social capital have an impact on academic results, even after controlling for family characteristics (DiMaggio, 1982).

Regarding school success and academic achievements, (Hatos, 2010a,b; Hatos and Bǎlţǎtescu, 2013) identified socioeconomic status, school engagement, and leisure style as individual-level predictors. The authors also identified classroom level – the proportion of students with fathers with higher education – and school-level predictors – average achievement of the school students and school type. As the author explained, school-level variables account for the largest variance (Hatos, 2010b). In this sense, the differences between schools concerning students' backgrounds and teachers correlate with educational achievements. Consequently, students with lower grades are nested together with low satisfaction with regard to school and lower socioeconomic status. These groups are mostly composed of boys (Hatos, 2010b).

Welsh et al. (1999) present evidence of school deviance predictors at multiple levels, namely, age, race, gender, school involvement, belief in rules, and positive peer association at the individual level and school size, student perception of school climate, and school at the community level. A recently published analysis (Dullas et al., 2021) shows that adolescent boys are more likely to engage in delinquent behavior or more serious deviant acts, while adolescent girls are more often perpetrators of minor deviant acts.

#### Internet and Computer Use

Lee (2018) identifies two main theoretical frameworks that explain the occurrence of online deviant behaviors: self-control and social learning theory. Rooted in the general theory of crime, self-control theory asserts that individuals with low-level control have a higher chance of engaging in online deviant acts such as computer piracy, online exposure to sexually explicit materials, online harassment, and cyber deviance in general (Holt et al., 2012, 2015). Moreover, teenagers who have a higher sensation-seeking attitude have a positive attitude toward risk-taking and most often indulge in illegal downloading of music, games, and sexual content (Weisskirch and Murphy, 2004). Deviant peer affiliation partially mediates the link between adolescent sensation seeking and internet gaming addiction (Tian et al., 2019).

Association with deviant peers, including online interaction with virtual peers, is the main predictor of cyber-deviance (Bossler and Holt, 2009; Burgess-Proctor et al., 2009; Bossler et al., 2011; Holt et al., 2012; Lee, 2018). Subcultural theorists explain the mechanisms through which cyberspace assists in justifying engagement in deviant acts such as computer hacking and digital piracy, transferring social relationships from offline to online, and creating communities and shared norms (Holt, 2007, 2020; Holt and Copes, 2010; McCuddy and Esbensen, 2020).

Rooted in Bourdieu and Weber's theories, the theory of digital divide holds that the Internet amplifies the existent social inequalities, with people lacking digital skills (second-level digital divide) and the opportunities for making effective use of them if they acquired (third-level digital divide) left behind (DiMaggio and Hargittai, 2001; DiMaggio et al., 2001; Van Dijk and Hacker, 2003; DiMaggio and Garip, 2012; Barbovschi and Balea, 2013; Van Deursen and Van Dijk, 2015; Van Deursen et al., 2015; Scheerder et al., 2017; Hatos, 2019; Van Dijk, 2020).

Computer proficiency and technology use correlate with cyber deviant acts, including hacking, digital piracy, and online harassment (Lee, 2018). Therefore, we can conclude that the digital world is a field where actors attempt to gain social, symbolic, and digital capital to ensure and justify their power (Ragnedda and Muschert, 2013; Lindell, 2017; O'Neil and Ackland, 2020).

## Discussion

Starting from the question how the topics of deviance and cyber-deviance are covered in social sciences, the present review gathers the relevant findings on the field in order to create a comprehensive account of the phenomenon. As we have presented in the results section, our systematic literature review involved the analysis of two databases, namely 61 sources in extenso (Dataset 1) and 488 abstracts (Dataset 2). We chose to review the 61 articles in extenso in order to understand in more detail the most common themes and the main predictors of deviance. These issues could not be reflected so accurately by only the abstract analysis. The articles in extenso give us detailed information with a smaller number of sources, and in addition, the extrapolation of the analysis to the database of 488 abstracts allows us a holistic understanding of the phenomenon of deviance. The analysis of a higher number of articles in dataset 2 also facilitated the inclusion of more recent articles that do not have a consistent number of citations yet, with articles addressing more specific aspects of deviance. It may be noticed that the most cited articles are those that deal with classical theories of deviance.

A more detailed assessment of the results of in extenso articles highlights that the main themes in the 61 most cited articles published in the Web of Science database on the topic of deviance among adolescents refer to the predictors of deviance (61 articles), as a category in which all predictors are included such as school education, socioeconomic status, as well as two important categories that also represent predictors but taken as separately.

By holistically examining the two datasets, we provided an overview of the field. As compared to other systematic reviews focused only on a specific field of deviance (McGrath et al., 2011; Brauer and Tittle, 2012; Longobardi and Badenes-Ribera, 2019; Estévez et al., 2020), we analyzed the deviance in a general manner. This was possible by using computational text analysis, method which allowed an accurate screening of nearly 500 sources. As related to this aspect, **a first objective** consisted of identifying the main topics approached in the literature on deviance. From our knowledge, this objective was not addressed by any other systematic reviews. This method revealed four latent clusters of topics: *explanatory* theories of the causes of deviance (social learning, social control, subcultural theories), *socio-constructionist* theories (labeling theory, power theory, conflict theory), *predictors of deviance* (gender, socioeconomic status, family influence, family background, peers' affiliation) and *online deviance* (frequency of Internet use, digital divide).

With regards to the objective of revealing the most frequently encountered themes, our review shows that the phenomenon of deviance is explained grounded on the classical theories. The researchers choose between *explanatory theories* such as: differential association, routine activities and opportunities theories, social learning, social control, social disorganization, anomie and strain theories, subcultural theories, power relations, and neutralization and deterrence theories (Benda and Corwyn, 1997; Wagner et al., 2004; Wallace et al., 2007; Dolliver and Rocker, 2017; Meldrum et al., 2020), or *socio-constructionist*- symbolic interactionism, labeling theory, phenomenological theory, critical discourse analysis, cultural theories, framing theory, convenience theory, and post-modernist theories (Giordano et al., 2009; Herman-Kinney and Kinney, 2013; Gottschalk, 2018, 2020; Barmaki, 2021) or even the integrative ones, respectively social disorganization theory and life-course theories (Apel and Sweeten, 2010; Peguero, 2011; Payne and Welch, 2016; Gostjev and Nielsen, 2017). Consequently, our review showed that most of the analyzed studies centered on the *predictors and correlates of deviance*. It highlights their focus on revealing and characterizing the predictors of deviance, mainly using *strain theory* (Aseltine et al., 2000; Cheung and Cheung, 2010; Adamczyk, 2012; Bruno et al., 2012; Scheuerman, 2019), *social control* (Free, 1992; Woodward et al., 2001; Jang, 2002; Byrd et al., 2015), *social learning theories* (Barnes and Farrell, 1992; Benda, 1994; Winfree et al., 1994; Terrell, 1997; Regnerus, 2002), or routine opportunity theories (Osgood et al., 1996; Marcum et al., 2010; Maimon and Browning, 2012b; Ragan et al., 2014; Yuan and McNeeley, 2018). As we observed from the manual analysis of the 488 articles, this is mainly done through quantitative analysis; 348 articles out of 488 were examined using quantitative methods. Furthermore, there is a significant concern for *online deviance and online deviant behaviors* (Marcum et al., 2010; Oksanen et al., 2014; Rafalow, 2015; Vejmelka et al., 2017; Mesch, 2018; Tomczyk, 2018; Chester et al., 2019; Granic et al., 2020; Semenza, 2021). The extensive number of identified studies focused on online deviance and adolescents is connected to the popularity of Internet among teenagers and compulsive use of it (Wachs et al., 2015; Kapoor et al., 2017; Ohannessian and Vannucci, 2020). In addition, with the spread of Internet technology, teenagers' online deviant behavior has become a matter of grave concern for parents, educators, social workers and researchers.

As related to the **second objective**, we observed an overlap between psychological and sociological approaches, with researchers making use of predictors at individual and social levels to explain deviance. Hence, theories such as differential reinforcement, social learning, problem-behavior, social bond theory, self-control theory, rational choice theory, differential social support, terror management theory, theories of maturation, and psychanalytic theories represent interdisciplinary perspectives that are of interest both for sociologists and psychologists. At the same time, our research revealed theories developed both by sociologists and criminologists to explain the nature of deviant behavior, including differential association, routine activities and opportunities theories, social control, social disorganization, anomie and strain theories, subcultural theories, power relations, and neutralization and deterrence theories. In addition, the dataset contained articles concerning the social construction of deviance, encompassing theories, such as: symbolic interactionism, labeling theory, phenomenological theory, critical discourse analysis, cultural theories, framing theory, convenience theory, and post-modernist theories.

Consistent to the literature, our review identified five main categories of *predictors* (**the third objective** addressed) that influence the engagement of teenagers in online and offline deviant behaviors, namely: family patterns, socio-demographic aspects, victimization, school and individual factors, Internet and computer use. The most employed predictors for explaining deviance include: family characteristics, family background, peer affiliation, school factors, school results, religion, risk attitude, religion, risk factors, victimization (Dukes and Lorch, 1989; Bahr et al., 1993; Aseltine, 1995; Benda and Corwyn, 1997; Amato and Fowler, 2002; Bjarnason et al., 2005; Haynie and Osgood, 2005; Chapple et al., 2014; Corkin et al., 2015; Buehler, 2020). At the same time, most of the longitudinal studies that we analyzed deal with the topic of delinquency follow a life-course perspective (Uggen and Kruttschnitt, 1998; Macmillan, 2001; Giordano et al., 2002; Kirk and Sampson, 2013; Salvatore and Markowitz, 2014; Pratt et al., 2016). As the papers analyzed show, online deviance has similar predictors with offline deviance, fact that suggests a continuity between the two. Still, the engagement into online deviance has some specific predictors, such as: frequency of internet use, patterns of internet use, computer skills, with digital divide theory as a main paradigm (Broos and Roe, 2006; Hinduja and Patchin, 2008; Clark, 2009; DiMaggio and Garip, 2012; Lindsay and Krysik, 2012; Chen et al., 2017; Chan and Wong, 2019a,b). Moreover, the articles analyzed on online deviance focus on the influence of lifestyle and the issue of identity (Zhao, 2005; Robinson, 2009; Karaian, 2012; Harvey et al., 2013; Barmaki, 2021).

Taking everything into consideration, our results show that social scientists employ classical theories of deviance for understanding the nature of the phenomenon and are particularly concerned with identifying the predictors and the interactions between them as related to online and offline deviance.

### Limitations

The results of this study need to be interpreted with caution. In this sense, we recommend future researchers conduct more in-depth systematic text review analyses based on other search queries that permit the identification of relevant articles on the study of deviance and cyber-deviance in the period of adolescence. Considering the present research, we state some limits related to the number of analyzed texts, code creation, and interpretation and computational constraints. First, in the last stage of analysis, we decided to select only the articles that responded to the second and third criteria as the number of articles that fit the first criterion was consistently large. We chose to use this approach because of computational limits as employing text searches on 488 articles exceeds the capacity of our computer memory.

Considering that for the last two criteria, the in extenso articles were selected only on the basis of the number of citations; the studies selected in the first dataset include a higher number of classical sources and, therefore, possibly excluded some recent impact articles.

Another limit relates to subjective code creation. However, the data was automatically clustered for diminishing the potential bias. The revealed clusters were compared to the data that are classified based on the designed codes.

### Conclusion

This paper presents a systematic review of the literature on the phenomenon of online and offline adolescent deviance grounded in the main findings reported in the studies published in the Web of Science database until April 2021.

For the analysis, we decided to use a validated methodology – the systematic quantitative literature review method described by Pickering and Byrne (2014), with additional tools from the PRISMA model. For reliability and systematic inquiry, the reviews were performed using the KH Coder software.

Our systematic literature review provides a clearer picture of adolescent deviance. At the same time, the differences and overlaps among the sociological and psychological approaches to the phenomenon were clarified. Psychologists and sociologists often take an interdisciplinary approach to explain the phenomenon of deviance. The most employed theories consist of strain theory, social learning, self-control, and social control theories.

The results of the research analyzed showed that the most frequently encountered themes in the area of deviance, can be grouped in four main topics: predictors of deviance, online deviance, socio-constructivist theories, and explanatory theories of the nature of deviant behaviors.

The present systematic literature review emphasized that the main categories of predictors in the case of deviance consist of family patterns, socio-demographic aspects, socialization, victimization, and school and individual factors. The study of online deviance needs to consider the existing digital divides. Along with the digital divide, the study of online behavior must examine the types of online capital and their interdependence with the offline world. Specific to online deviance, in addition to the predictors related to the above-mentioned offline context, the use of the internet and computer skills also have a significant impact in explaining the deviance phenomenon.

The paper asserts that deviance and online deviance are interrelated phenomena that can be explained by distinct theoretical approaches. As the study may show, the online sphere does not represent just the medium where activities or behaviors take place. Rather, it deepens the existing social inequalities, amplifying the differences attributed to social status. Moreover, deviance in the online environment is mostly studied in relation to children, teenagers, and youth, since the Internet is quite popular among these age groups.

We believe that the present systematic literature review makes an important contribution to the understanding of deviance by presenting an overview of the phenomenon. The findings of this literature review are useful for experts in the fields of psychology, sociology, and other related fields. The methods and software used for source analysis (Pickering and KH Coder) allowed us to identify the most relevant predictors of deviance and the most common themes for approaching the phenomenon. Our methods may also be applied to the research of experts of other fields who are interested in studying a specific phenomenon, as it allows a significant number of sources to be included in the analysis.

## Data Availability Statement

The original contributions presented in the study are included in the article/Supplementary Material, further inquiries can be directed to the corresponding author.

## Author Contributions

SC and AH were responsible for designing the research. AH and CB supervised the data analysis and the manuscript writing. SC contributed to the data coding and analysis. SC and AL wrote the first draft of this manuscript. CB and AL contributed to the conceptualization of the study, to editing, and to revising the manuscript. All authors listed have made a substantial, direct and intellectual contribution to the work, and approved it for publication.

## Funding

This work was partial funded by Project 123008, SmartDoct – High quality programs for doctoral and post-doctoral students of Oradea University, for the increase of relevance in research and innovation, in the context of the regional economy, project financed by Human Capital Operational Program 2014-2020.

## Conflict of Interest

The authors declare that the research was conducted in the absence of any commercial or financial relationships that could be construed as a potential conflict of interest.

## Publisher's Note

All claims expressed in this article are solely those of the authors and do not necessarily represent those of their affiliated organizations, or those of the publisher, the editors and the reviewers. Any product that may be evaluated in this article, or claim that may be made by its manufacturer, is not guaranteed or endorsed by the publisher.

## Footnotes

^1**^ < 0.01 ^*^ < 0.05.
