# Supplementary figures and images for: Adolescent Deviance and Cyber-Deviance. A Systematic Literature Review
Source: Front Psychol. 2021 Oct 12;12:748006. doi: 10.3389/fpsyg.2021.748006 (PMC8546304; doi:10.3389/fpsyg.2021.748006)

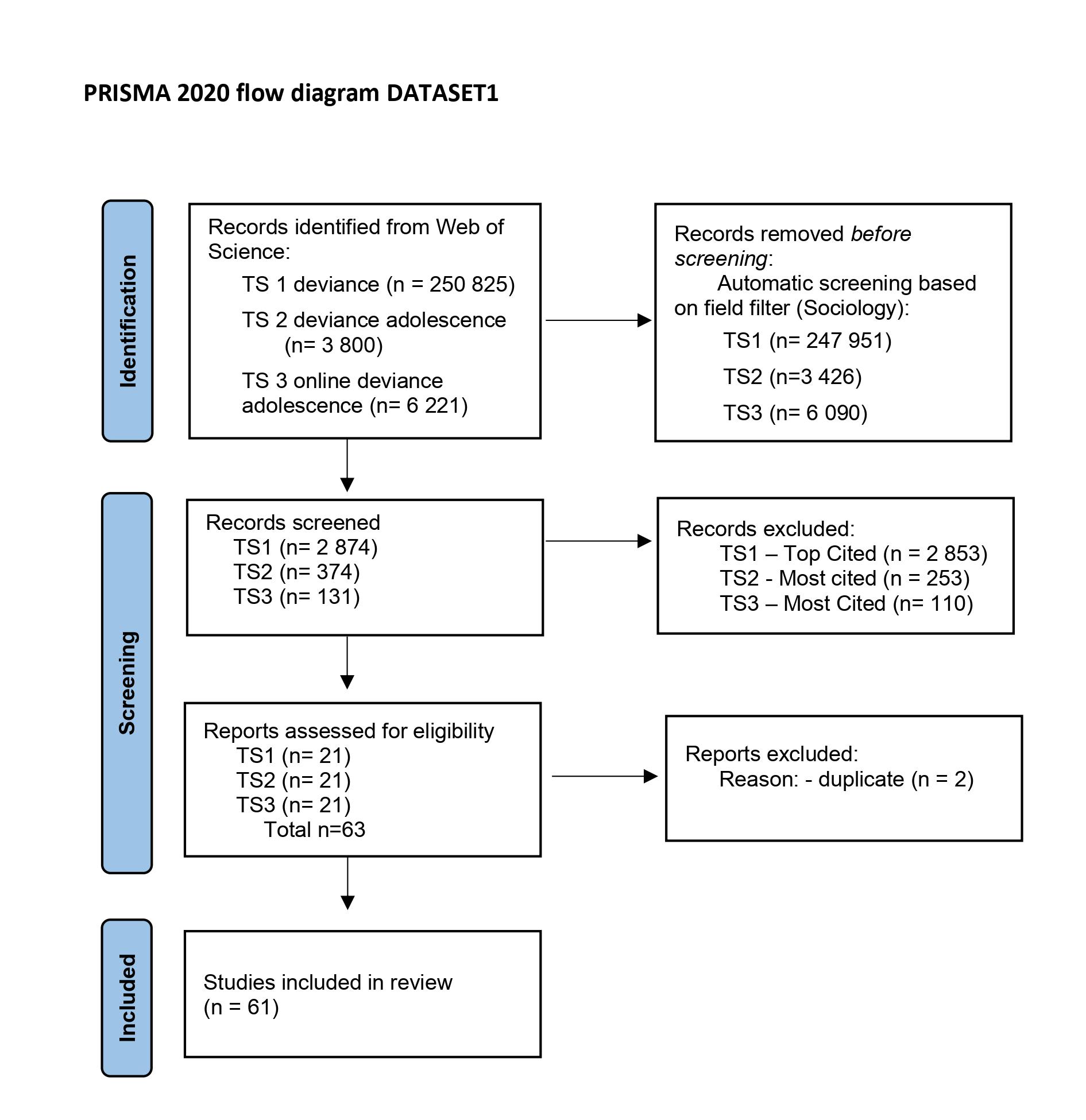

Supplement: Supplementary file 1 [file Image_1.jpg]

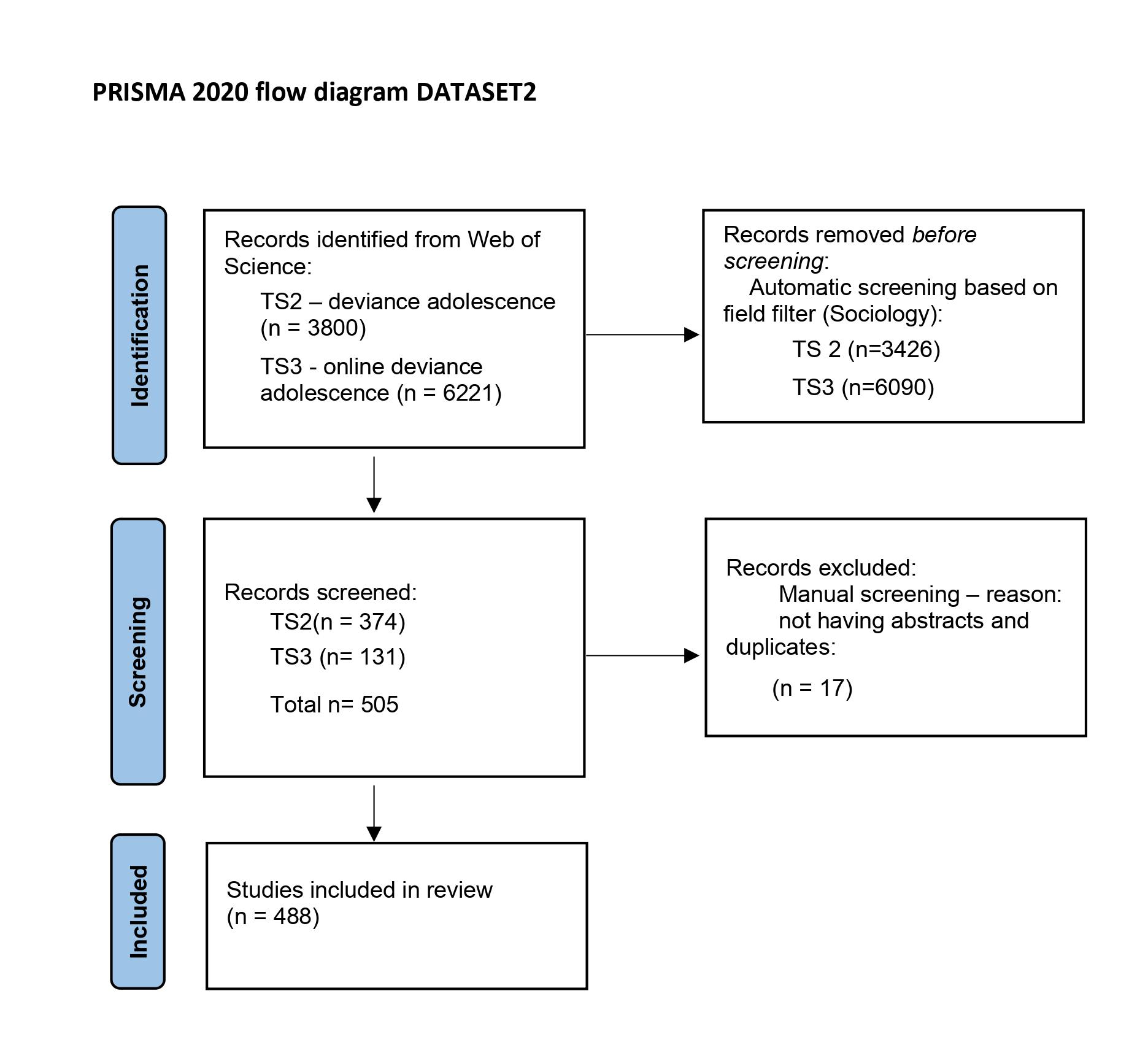

Supplement: Supplementary file 2 [file Image_2.jpg]
